# *Akkermansia muciniphila* attenuates intervertebral disc degeneration via extracellular vesicle-mediated delivery of the effector protein B2UKX5

**DOI:** 10.1038/s41413-026-00541-5

**Published:** 2026-05-20

**Authors:** Zhe Guan, Xiaoxue Li, Yixiao Chen, Sheng Zhu, Jie Wen, Hongliang Zhou, Chunyuan Chen, Jianghua Liu, Guoqiang Zhu, Zhilin Pang, Yiwei Liu, Ling Jin, Shiyu Zeng, Yi Luo, Xiaoxiao Gong, Yu Yang, Ya Chen, Yang Wu, Meidan Wan, Hao Yin, Yong Zhou, Zhenxing Wang, Hui Xie

**Affiliations:** 1 Department of Orthopedics, Movement System Injury and Repair Research Center, Xiangya Hospital, Central South University, Changsha, Hunan China; 2 Hunan Key Laboratory of Angmedicine, Changsha, Hunan China; 3 Department of Gastroenterology, The First Affiliated Hospital of Harbin Medical University, Harbin, Heilongjiang China; 4 Department of Neurosurgery, Xiangya Hospital, Central South University, Changsha, Hunan China; 5 The First Affiliated Hospital of University of South China, Hengyang, Hunan China; 6 Department of Respiratory Medicine, National Key Clinical Specialty, Branch of National Clinical Research Center for Respiratory Disease, Xiangya Hospital, Central South University, Changsha, Hunan China; 7 Department of Neurology, Xiangya Hospital, Central South University, Changsha, Hunan China; 8 Third Xiangya Hospital, Central South University, Changsha, Hunan China; 9 National Clinical Research Center for Geriatric Disorders, Xiangya Hospital, Changsha, Hunan China

**Keywords:** Metabolism, Endocrine system and metabolic diseases

## Abstract

Low back pain is a leading cause of global disability, with intervertebral disc degeneration (IVDD) as a primary contributor. Emerging evidence suggests a link between gut microbiota and disc health, yet the underlying mechanisms remain unclear. Through Mendelian randomization and a clinical cohort analysis, we identified a causal inverse relationship between *Akkermansia muciniphila* (*Akk*) abundance and IVDD risk, with reduced fecal *Akk* levels correlating with increased IVDD severity. *Akk* protected against IVDD in microbiota-depleted mice, and this protection was abolished by pharmacologic inhibition of extracellular vesicle (EV) secretion. Consistently, *Akk*-derived EVs (*Akk*-EVs) recapitulated the benefits of *Akk* across natural aging, tail needle puncture, and bipedal standing mouse models, while control bacterium (*Escherichia coli*) and its EVs did not. Proteomics and functional validation identified B2UKX5 as a key *Akk*-EV-enriched effector protein. Furthermore, recombinant B2UKX5 attenuated IVDD in vivo and regulated critical pathways for disc homeostasis, including collagen synthesis, extracellular matrix remodeling, and chromatin silencing, as revealed by transcriptomic profiling of microdissected nucleus pulposus and annulus fibrosus tissues. Analysis of clinical samples further confirmed that *Akk*-EVs and B2UKX5 levels in circulation and intervertebral disc tissues were negatively correlated with IVDD severity. These findings establish a novel gut-disc axis, highlighting *Akk*, *Akk*-EVs, and B2UKX5 as promising therapeutic candidates for IVDD prevention and treatment.

## Introduction

Low back pain is a leading cause of disability worldwide, with substantial impacts on both individual quality of life and the global economy.^1^ Intervertebral disc degeneration (IVDD) is a predominant contributor to low back pain, particularly in its early stages, where disc-mediated pain often drives the onset of symptoms.^2,3^ IVDD is characterized by a progressive, cell-mediated structural and functional deterioration of the intervertebral disc, driven by disrupted homeostasis in nucleus pulposus (NP) and annulus fibrosus (AF) cells.^4^ Known risk factors for IVDD include genetic predisposition, aging, mechanical overloading, smoking, and infections,^5–10^ which collectively promote cytokine release, catabolic enzyme activity, and extracellular matrix (ECM) breakdown.^11^ Current treatments remain largely symptomatic, relying on physical therapy, analgesics, or invasive procedures,^12^ and a definitive cure or preventive approach for IVDD progression remains elusive in clinical practice. Consequently, there is an urgent need for identifying novel preventive and therapeutic strategies for IVDD.

The gut microbiota exerts profound systemic influences on host physiology, including metabolism and immune function.^13^ Increasing evidence highlights bidirectional communication between the gut microbiota and multiple organ systems, such as gut-brain and gut-skeletal axes.^14^ Recent Mendelian randomization (MR) studies have suggested a causal association between gut microbiota composition and IVDD risk,^15–18^ with observational data revealing microbiota dysbiosis in patients with disc disease.^19^ Preliminary reports indicate that gut microbiota modulation may alleviate experimental IVDD,^20,21^ but the specific bacterial species and the mechanisms responsible remain undefined.

The complex pathogenesis of IVDD is characterized by chronic inflammation, oxidative stress, and metabolic dysregulation.^22,23^ In this context, the gut microbiota has emerged as a potential systemic modulator, particularly through the proposed gut-disc axis. Among the gut microbiota, *Akkermansia muciniphila* (*Akk*), an abundant intestinal commensal, has been shown to play a crucial role in regulating inflammation, metabolism, and intestinal barrier integrity.^24^ Despite these established functions, the potential relationship between *Akk* and IVDD remains largely unexplored, prompting a fundamental investigation into whether this microbial species can influence IVDD progression.

*Akk* secretes various metabolic products, including extracellular vesicles (EVs), short-chain fatty acids, and immunomodulatory factors, which have been implicated in modulating host health.^25,26^ Notably, *Akk*-derived EVs (*Akk*-EVs) have demonstrated protective effects in models of obesity, colitis, and hypertension.^27–29^ Our previous research has revealed the osteoprotective properties of *Akk*-EVs, demonstrating their ability to maintain bone mass.^30^ Based on these observations, we hypothesized that *Akk* and *Akk*-EVs might exert protective effects on the intervertebral disc, potentially slowing IVDD progression through their bioactive components. The molecular cargo of EVs, comprising proteins, RNA species, genomic DNA, and various non-coding RNAs, is believed to mediate their biological effects.^31^ Identifying the key molecular components of *Akk*-EVs that regulate disc metabolism is critical for advancing translational research in IVDD.

Here, we employed MR analysis and clinical fecal cohort studies to establish the causal relationship between *Akk* abundance and IVDD risk in humans, with reduced fecal *Akk* levels correlating with greater disease severity. We then leveraged antibiotic-mediated microbiota depletion to show that *Akk* alleviates IVDD. Pharmacologic inhibition of *Akk*-EVs release by GW4869 abolished *Akk*-mediated disc-protective effects, implicating EVs as critical mediators. Building on these findings, we evaluated the therapeutic potential of *Akk*-EVs in three complementary IVDD models, including natural aging, tail needle puncture, and bipedal standing in mice, and found that *Akk*-EVs recapitulated the protective effects of *Akk*. Through proteomic analysis of *Akk* and *Akk*-EVs, followed by in vitro and in vivo validation, we identified the EV-enriched effector protein B2UKX5 as a key mediator of disc protection. Furthermore, we utilized microdissection to isolate the NP and AF regions for transcriptomic sequencing to elucidate the underlying mechanisms by which B2UKX5 influences disc metabolism. Finally, in human peripheral blood and degenerating NP samples, *Akk*-EV abundance and B2UKX5 levels were inversely correlated with IVDD severity. Together, this multi-tiered evidence links *Akk*, *Akk*-EVs, and B2UKX5 to disc homeostasis, and provides a mechanistic rationale for gut microbiota-derived strategies to mitigate or delay IVDD.

## Results

### MR and cohort validation support an inverse association between intestinal *Akk* abundance and IVDD

**Fig. 1:**
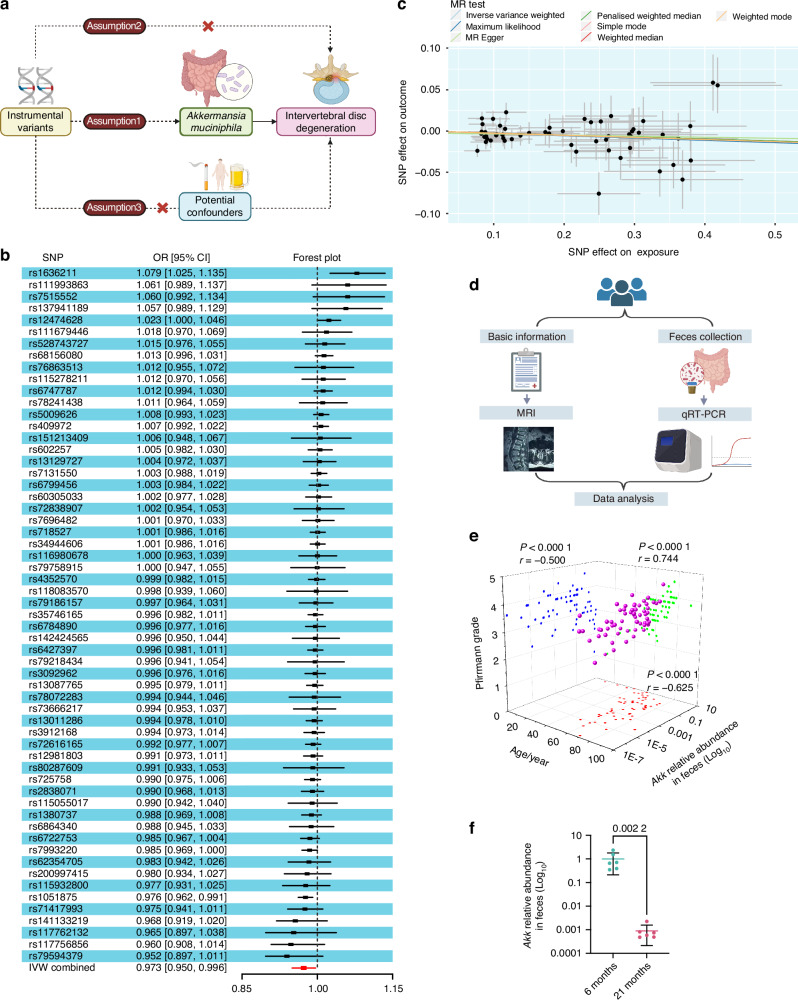
MR and cohort validation support an inverse association between intestinal *Akk* abundance and IVDD. **a** Schematic representation of the MR analysis workflow, including data sources, SNP selection criteria, and analysis pipeline. **b** Forest plot showing the effects of instrumental variables (IVs) on IVDD risk, with effect sizes and confidence intervals. **c** Scatter plot comparing MR results from multiple methods. **d** Schematic representation of the clinical sample collection protocol for human subjects. **e** Correlation analysis between age, IVDD Pfirrmann grade, and *Akk* relative abundance in human feces, with Pearson correlation coefficients indicated. *n* = 62. **f** qRT-PCR analysis of fecal *Akk* abundance in 6- and 21-month-old mice. *n* = 6 per group. Data are presented as mean ± SD. Statistical analysis was determined by Pearson correlation (**e**) or an unpaired Student’s *t*-test (**f**). OR odds ratio, CI confidence interval

To investigate whether genetically proxied intestinal abundance of *Akk* influences the risk of IVDD, we performed MR analysis using genetic proxies to estimate *Akk* abundance. The analysis workflow was illustrated in Fig. 1a. A total of 59 single nucleotide polymorphisms (SNPs) were identified and utilized as instrumental variables (IVs) to represent genetically determined *Akk* levels in the gut microbiota (Fig. 1b). Inverse variance weighting (IVW), the primary MR method, identified a significant negative association between higher *Akk* abundance and IVDD risk [odds ratio: 0.973, 95% confidence interval: 0.950–0.996, *P* = 0.02], suggesting a protective effect of *Akk* against IVDD (Fig. 1b, c). The consistency of this association was further validated by multiple MR methods, including maximum likelihood estimation, MR-Egger regression, penalized weighted median, simple mode, weighted median, and weighted mode, all of which supported the IVW results (Fig. 1c).

Sensitivity analyses confirmed the reliability of these findings. The funnel plots and Cochran’s Q test showed no evidence of heterogeneity among SNPs (*P* > 0.05, Fig. S1a), and the leave-one-out analyses confirmed that no single SNP disproportionately influenced the results (Fig. S1b). Moreover, the MR-Egger intercept test revealed no evidence of horizontal pleiotropy (*P* > 0.05), supporting the absence of confounding factors. Together, these results suggest a potential causal relationship between higher intestinal *Akk* abundance and reduced IVDD risk.

To corroborate the MR findings with direct observational evidence, we quantified fecal *Akk* relative abundance by quantitative real-time PCR (qRT-PCR) in a clinical cohort of 62 patients (aged 12-91 years) with MRI-graded IVDD using the Pfirrmann classification. The sample collection and processing protocol is outlined in Fig. 1d, with patient characteristics detailed in Table S1. Correlation analyses demonstrated a significant inverse relationship between fecal *Akk* abundance and Pfirrmann grade (*P* < 0.000 1, *r* = −0.500) as well as patient age (*P* < 0.000 1, *r* = −0.625), while age positively correlated with IVDD severity (*P* < 0.000 1, *r* = 0.744; Fig. 1e).

Given the established role of aging in IVDD progression,^7,32^ we subsequently examined the age-dependent abundance of *Akk* in naturally aging mice.^33,34^ Consistent with the population-level findings, *Akk* abundance was significantly lower in the feces of 21-month-old mice compared to 6-month-old counterparts (Fig. 1f).

Taken together, these validation experiments in human fecal samples and aging mice are consistent with the protective association suggested by our MR analysis. Furthermore, the observed reduction in *Akk* levels with aging was consistent with previous reports indicating gut microbiota dysbiosis in aged individuals,^30,35^ thereby further supporting the hypothesis that *Akk* may contribute to maintaining intervertebral disc health.

### *Akk* supplementation alleviates IVDD in microbiota-depleted mice

**Fig. 2:**
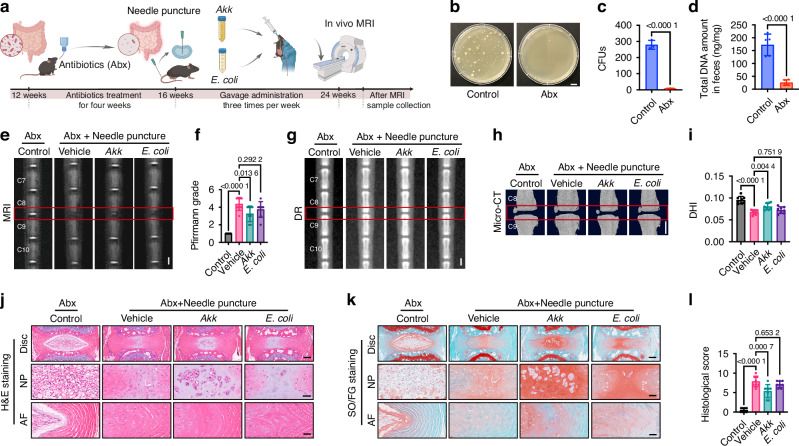
*Akk* supplementation alleviates IVDD in microbiota-depleted mice. **a** Schematic of the experimental protocol for *Akk* and *E. coli* treatment in the antibiotic-depleted, needle-puncture IVDD model. **b** Representative LB agar plates images showing bacterial colonies derived from fecal supernatants of control and antibiotic-treated mice. Scale bar: 1 cm. **c** Quantification of CFUs cultured from fecal supernatants on LB agar plates. *n* = 3 per group. **d** Quantitative analysis of total DNA extracted from fecal samples. *n* = 5 per group. **e** Representative T2-weighted MRI images of the C8-9 caudal intervertebral disc. Scale bar: 1 mm. **f** Statistical analysis of Pfirrmann grading for the C8-9 caudal segment. *n* = 8 per group. Representative DR images (**g**) and micro-CT 3D reconstruction images (**h**) of the caudal spine. Scale bar: 1 mm. **i** DHI for the C8-9 caudal segment. *n* = 8 per group. Representative H&E (**j**) and SO/FG (**k**) stained images showing the macroscopic morphology of the disc, NP, and AF in the C8-9 segment. Scale bars: 200 μm (Disc) or 50 μm (NP, AF). **l** Histological scores for the C8-9 segment. *n* = 8 per group. Data are presented as mean ± SD. Statistical significance was determined by unpaired Student’s *t*-test (**c**, **d**) or one-way ANOVA followed by Bonferroni post hoc test (**f**, **i**, **l**)

To exclude confounding effects from the endogenous gut microbiota, we generated microbiota-depleted mice using a broad-spectrum antibiotic cocktail (Abx).^36^ After Abx treatment, IVDD was induced using the widely used tail disc needle puncture model, followed by oral gavage of vehicle (PBS), *Akk*, or *Escherichia coli* (*E. coli*, a commensal control strain), with non-punctured Abx-treated mice serving as baseline controls (Fig. 2a). Depletion efficiency was confirmed by markedly reduced colony-forming units (CFUs) from fecal suspensions on agar plates and lower fecal bacterial DNA yield in Abx mice relative to untreated controls (Fig. 2b–d).

At the experimental endpoint, T2-weighted MRI revealed robust puncture-induced degeneration in Abx-treated vehicle mice compared with baseline controls, manifested by reduced T2 signal intensity (Fig. 2e, f). Notably, *Akk*-treated punctured mice significantly preserved T2 signal, indicating attenuated degeneration (Fig. 2e, f). Consistently, digital radiography (DR) and micro-computed tomography (micro-CT) analyses showed that Abx-treated vehicle mice exhibited a marked reduction in disc height index (DHI) compared with baseline controls, whereas *Akk* significantly maintained DHI (Fig. 2g–i). Histological analyses, including H&E and Safranin-O/Fast green (SO/FG), revealed canonical degenerative features in Abx-treated vehicle discs relative to baseline controls, including loss of NP cellularity, ECM depletion, and disruption of the annulus fibrosus (AF). In contrast, *Akk* significantly preserved NP cellularity and ECM organization, together with a reduction of AF fissures (Fig. 2j–l). However, *E. coli* gavage failed to improve MRI, DHI, or histopathological outcomes across these readouts (Fig. 2e–l).

Together, these data indicate that *Akk* can alleviate IVDD under antibiotic-mediated microbiota depletion.

### *Akk* protects against IVDD via EV-dependent mechanisms

Although *Akk* has been reported to communicate with the host through several bioactive components, including metabolites and EVs, direct experimental evidence identifying the key mediator of its disc-protective activity remains lacking. To determine whether *Akk*’s protection against IVDD depends on EV secretion, we employed GW4869, a widely used pharmacological inhibitor of neutral sphingomyelinase 2, which has been reported to suppress bacterial EV release.^30,37,38^

**Fig. 3:**
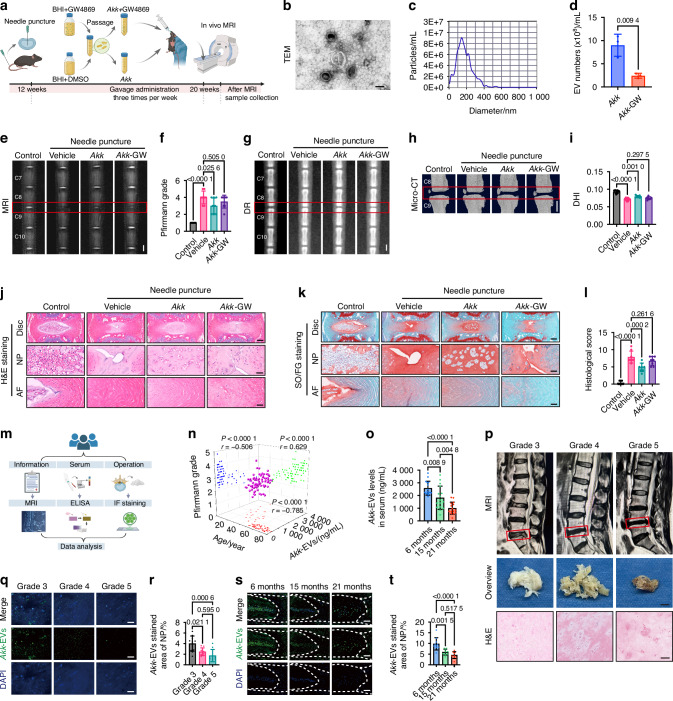
*Akk* protects against IVDD via EV-dependent mechanisms. **a** Schematic of the experimental protocol for *Akk* and *Akk*-GW4869 treatment in the needle puncture IVDD model. **b** Representative transmission electron microscopy image of *Akk*-EVs. Scale bar: 100 nm. **c** NTA of *Akk*-EVs showing the particle size distribution. **d** Quantitative NTA-based comparison of particle numbers extracted from the supernatants of *Akk* and *Akk*-GW4869 cultures. *n* = 3 per group. **e** Representative T2-weighted MRI images of the C8-9 caudal spine. Scale bar: 1 mm. **f** Statistical analysis of Pfirrmann grading for the C8-9 caudal segment. *n* = 8 per group. Representative DR images (**g**) and micro-CT 3D reconstruction images (**h**) of the caudal spine. Scale bar: 1 mm. **i** DHI for the C8-9 caudal segment. *n* = 8 per group. Representative H&E (**j**) and SO/FG (**k**) stained images showing the macroscopic morphology of the disc, NP, and AF in the C8-9 segment. Scale bars: 200 μm (Disc) or 50 μm (NP, AF). **l** Histological scores for the C8-9 segment. *n* = 8 per group. **m** Schematic representation of the clinical sample collection protocol for human subjects. **n** Correlation analysis between age, IVDD Pfirrmann grade, and serum *Akk*-EVs levels in human subjects, with Pearson correlation coefficients indicated. *n* = 79. **o** Quantitative analysis of *Akk*-EVs levels in serum from mice of different age groups. *n* = 15–16 per group. **p** Representative lumbar MRI scans, intraoperative NP tissue images, and H&E-stained sections of NP tissue from human subjects with different IVDD grades. The red rectangular box highlights the surgical lesion segment of the lumbar spine on MRI. Scale bars: 5 mm (for overview) or 100 μm (for H&E staining). **q** Representative IF staining of *Akk*-EVs (green) in human NP tissue from patients with varying degrees of IVDD; Nuclei are counterstained with DAPI (blue). Scale bar: 50 μm. **r** Quantification of *Akk*-EVs-positive area (%) in human NP tissue; *n* = 9–11 per group. **s** Representative IF staining of *Akk*-EVs (green) in intervertebral disc sections from young versus aged mice; Nuclei are counterstained with DAPI (blue). Scale bar: 100 μm. **t** Quantification of *Akk*-EVs-positive area (%) in mouse discs. *n* = 9–10 per group. Data are presented as mean ± SD. Statistical significance was determined by unpaired Student’s *t*-test (**d**), Pearson correlation (n) or one-way ANOVA followed by Bonferroni post hoc test (**f**, **i**, **l**, **o**, **r**, **t**)

In a needle puncture-induced IVDD model, mice received oral gavage of vehicle, *Akk*, or GW4869-pretreated *Akk* (*Akk*-GW; Fig. 3a). Isolated *Akk*-EVs displayed the characteristic cup-shaped morphology under transmission electron microscopy (Fig. 3b) and a typical size distribution by nanoparticle tracking analysis (NTA; Fig. 3c). Quantitative analysis further confirmed a marked reduction in EV yield in the *Akk-*GW group compared with the *Akk* group (Fig. 3d), validating effective inhibition.

Following the 8-week intervention, compared with vehicle, *Akk* treatment preserved T2 signal intensity (Fig. 3e, f), maintained DHI (Fig. 3g–i), and improved histopathological features, including reduced NP cell loss, ECM depletion, and AF disruption (Fig. 3j–l). In contrast, GW4869 pretreatment largely abolished these protective effects, with the *Akk*-GW group showing no significant improvement over vehicle across MRI, DHI, and histology (Fig. 3e–l). *Akk* treatment did not significantly alter serum levels of IFN-γ, TNF-α, interleukin (IL)-6, IL-2, or IL-10 compared with vehicle (Fig. S2), suggesting that protection is not driven by broad systemic inflammatory changes. Collectively, these results indicate that EV secretion is required for the disc-protective effects of *Akk*.

Next, we examined whether endogenous *Akk*-EV abundance associates with IVDD severity. We quantified serum *Akk*-EV levels by competitive enzyme-linked immunosorbent assay (ELISA) from 79 MRI-graded IVDD patients (aged 18-79 years; workflow in Fig. 3m and detailed patient information in Table S2). The standard curve of ELISA is shown in Fig. S3a. Correlation analysis demonstrated a significant inverse relationship between serum *Akk*-EVs levels and Pfirrmann grade (*P* < 0.000 1, *r* = −0.506) and patient age (*P* < 0.000 1, *r* = −0.785), while age positively correlated with IVDD severity (*P* < 0.000 1, *r* = 0.629; Fig. 3n). Consistent with these findings, ELISA analysis of serum samples from naturally aging mice showed a progressive age-dependent decline in *Akk*-EVs levels (Fig. 3o).

Local *Akk*-EV abundance was assessed by immunofluorescence (IF) staining in human NP tissues from subjects aged 50–74 years with Pfirrmann grades of 3–5 (details in Table S2). Representative lumbar MRI scans, intraoperative gross images of NP tissue, and H&E stained sections are presented in Fig. 3p. IF analysis demonstrated that the *Akk*-EV-positive area decreased significantly with increasing degeneration severity (Fig. 3q, r). A similar age-dependent decline was also observed in NP sections from mice across age groups (Fig. 3s, t), paralleling the trend in human samples.

Taken together, the loss of *Akk* efficacy upon EV inhibition, coupled with the degeneration-associated reduction of endogenous *Akk*-EVs in serum and disc tissue, supports *Akk*-EVs as a potential key mediator through which *Akk* exerts disc-protective effects.

### Supplementation of *Akk* and *Akk*-EVs attenuates IVDD in multiple in vivo models

**Fig. 4:**
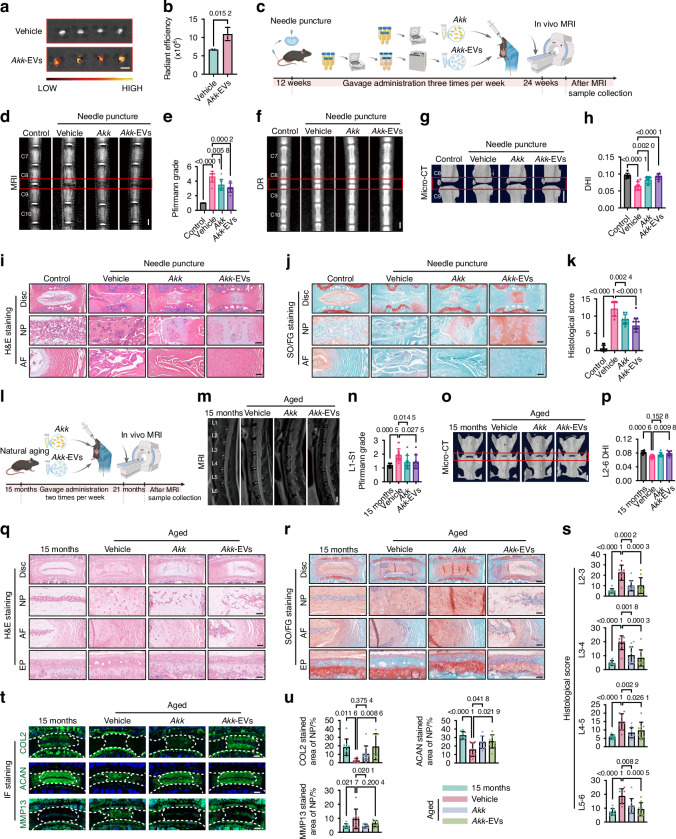
Supplementation of *Akk* and *Akk*-EVs attenuates IVDD in multiple in vivo models. **a** Representative ex vivo fluorescence images of L2-6 lumbar intervertebral discs 24 h after intravenous injection of DiR-labeled *Akk*-EVs in mice. Scale bar: 3 mm. **b** Quantitative analysis of mean fluorescence intensity across L2-6 in intervertebral discs. *n* = 3 per group. **c** Schematic of the experimental protocol for *Akk* and *Akk*-EVs treatment in needle puncture mice. **d** Representative T2-weighted MRI images of the C8-9 caudal spine. Scale bar: 1 mm. **e** Statistical analysis of Pfirrmann grading for the C8-9 caudal segment. *n* = 8 per group. Representative DR images (**f**) and micro-CT 3D reconstruction images (**g**) of the caudal spine. Scale bar: 1 mm. **h** DHI after 12 weeks of needle puncture at the C8-9 caudal segment. *n* = 8 per group. Representative H&E (**i**) and SO/FG (**j**) stained images showing the macroscopic morphology of the disc, NP, and AF in the C8-9 segment. Scale bars: 200 μm (Disc) or 50 μm (NP, AF). **k** Histological scores for the C8-9 segment. *n* = 8 per group. **l** Schematic of the experimental protocol for *Akk* and *Akk*-EVs treatment in naturally aging mice. **m** Representative T2-weighted MRI images of the lumbar spine in mice. Scale bar: 1 mm. **n** Statistical analysis of the average Pfirrmann grade in the L1-S1 lumbar disc segments. *n* = 9–14 per group. **o** Representative micro-CT 3D reconstruction images of the L5-6 lumbar disc segments. Scale bar: 1 mm. **p** Statistical analysis of the mean DHI in the L2-6 lumbar disc segments. *n* = 8–10 per group. **q**, **r** Representative images of H&E (q) and SO/FG (r) staining showing the macroscopic morphology of intervertebral disc (Disc), nucleus pulposus (NP), annulus fibrosus (AF), and endplate (EP) in the L5-6 lumbar disc segment. Scale bars: 200 μm (Disc) or 50 μm (NP, AF, EP). **s** Statistical analysis of histological scores for each lumbar segment (L2-6). *n* = 8–10 per group. **t** Representative IF images showing the expression of COL2, ACAN, and MMP13 in the L5-6 lumbar intervertebral discs. Scale bar: 200 μm. **u** Quantitative analysis of COL2, ACAN, and MMP13 expression. *n* = 8–10 per group. Data are presented as mean ± SD. Statistical significance was determined by unpaired Student’s *t*-test (**b**) or one-way ANOVA followed by Bonferroni post hoc test (**e**, **h**, **k**, **n**, **p**, **s**, **u**)

To test whether *Akk*-EVs act as functional mediators of disc protection in vivo, we first confirmed their in vivo biodistribution. DiR-labeled *Akk*-EVs were detectable in intervertebral discs, indicating that exogenously administered *Akk*-EVs can distribute to and accumulate within disc tissue (Fig. 4a, b), consistent with previous reports showing their localization in bone and other major organs.^30^

We then compared the therapeutic efficacy of *Akk*-EVs with *Akk* as a reference intervention across three complementary IVDD models representing distinct pathogenic drivers: tail needle-puncture (acute injury or inflammation),^39–41^ natural aging (progressive age-associated degeneration),^34,42^ and bipedal standing (mechanical overload).^43–45^

In the tail needle puncture model, mice received vehicle (PBS), *Akk*, or *Akk*-EVs by oral gavage three times per week for 12 weeks post-injury (Fig. 4c). At the experimental endpoint, T2-weighted MRI showed marked hypointensity in vehicle-treated discs, whereas both *Akk* and *Akk*-EVs partially preserved T2 signals (Fig. 4d, e). DR and micro-CT further demonstrated significant maintenance of DHI in both groups (Fig. 4f–h). Histological staining (H&E and SO/FG) and scoring showed preserved NP cellularity and matrix structure with reduced AF fissures in both groups (Fig. 4i–k). We further assessed ECM markers, including type II collagen (COL2) and aggrecan (ACAN), as well as the catabolic enzyme matrix metalloproteinase 13 (MMP13). Both *Akk* and *Akk*-EVs tended to preserve COL2 and ACAN while significantly reducing MMP13 (Fig. S4). In contrast, oral gavage of *E. coli*-EVs conferred no detectable protection across MRI, DHI, or histological readouts (Fig. S5). Consistent with the gavage results, intravenous administration yielded similar results: intravenous administration of *Akk*-EVs conferred robust benefits without systemic cytokine elevation, whereas *E. coli*-EVs were ineffective and induced splenomegaly with marked cytokine increases (Fig. S6).

In the natural aging model, 15-month-old mice were orally administered either vehicle (PBS), *Akk*, or *Akk*-EVs twice a week for 6 months, with untreated 15-month-old mice as baseline controls (Fig. 4l). *Akk* supplementation achieved stable gut colonization (Fig. S7) and elevated *Akk*-EV levels in gut lumen, serum, and disc tissues, with the highest levels in the *Akk*-EVs treatment group (Fig. S8). At the experimental endpoint, MRI showed that both *Akk*- and *Akk*-EVs-treated mice exhibited notable preservation of T2 signal intensity, as reflected by lower mean Pfirrmann grades across lumbar levels (L1-S1) than vehicle-treated mice (Fig. 4m, n). Micro-CT of L2-6 segments demonstrated significant maintenance of mean DHI in both treatment groups (Fig. 4o, p). Histological analyses of L2-6 lumbar discs showed that, compared with baseline controls, vehicle-treated discs exhibited marked NP cell loss and fibrotic remodeling, with disrupted AF architecture and blurring of the NP-AF boundary, accompanied by endplate ossification and fissures (Fig. 4q, r). Relative to vehicle, both *Akk* and *Akk*-EVs preserved NP cellularity and overall disc architecture, reduced rounded AF cells and fibrosis, and maintained clearer NP-AF boundaries (Fig. 4q, r). Accordingly, degeneration scores across L2-6 were significantly lower in the *Akk* and *Akk*-EVs groups than in the vehicle group (Fig. 4s). At the molecular level, both *Akk* and *Akk*-EVs preserved key extracellular matrix markers, including COL2 and ACAN, and reduced the catabolic enzyme MMP13 (Fig. 4t, u). Neither *Akk* nor *Akk*-EVs significantly altered endplate porosity or the number of osteoblasts/osteoclasts (Fig. S9), suggesting that these treatments do not substantially affect endplate structural remodeling in aging mice. Within the disc, local senescence (p16^Ink4a^) and inflammation markers (IL-6, TNF-α) were reduced (Fig. S10a, b). Systemic inflammatory cytokines and serum biochemistry were not altered, and major organs showed only age-related changes, with no evidence of additional *Akk*- and *Akk*-EV-related toxicity (Fig. S10c–e).

In the bipedal standing model, 12-week-old mice underwent a 1-week training period, followed by 12 weeks of bipedal standing, and received oral administration of vehicle, *Akk*, or *Akk*-EVs twice weekly throughout the intervention (Fig. S11a). Micro-CT showed a marked reduction in mean DHI across L2-5 segments in vehicle-treated mice compared with the baseline controls, whereas both *Akk* and *Akk*-EVs significantly maintained DHI (Fig. S11b, c). Histological analysis revealed NP compression, AF fissures, and endplate ossification in the vehicle group, while both *Akk* and *Akk*-EVs attenuated these degenerative changes (Fig. S11d, e). Consistent with these observations, immunofluorescence staining showed preserved COL2 and ACAN signals with reduced MMP13 in both treatment groups (Fig. S11f, g), supporting their ability to maintain extracellular matrix integrity under mechanical loading.

Taken together, across three complementary IVDD models driven by acute injury, aging, or mechanical overload, both *Akk* and *Akk*-EVs consistently delayed disc degeneration. Moreover, *Akk*-EVs recapitulated the disc-protective phenotype observed with *Akk*. Notably, long-term administration did not elicit systemic inflammatory activation or organ toxicity under our conditions. These findings support *Akk*-EVs as primary mediators of *Akk*-mediated disc protection and highlight their therapeutic potential for etiologically distinct forms of disc degeneration.

### B2UKX5 as a potential functional protein in *Akk*-EVs against IVDD

**Fig. 5:**
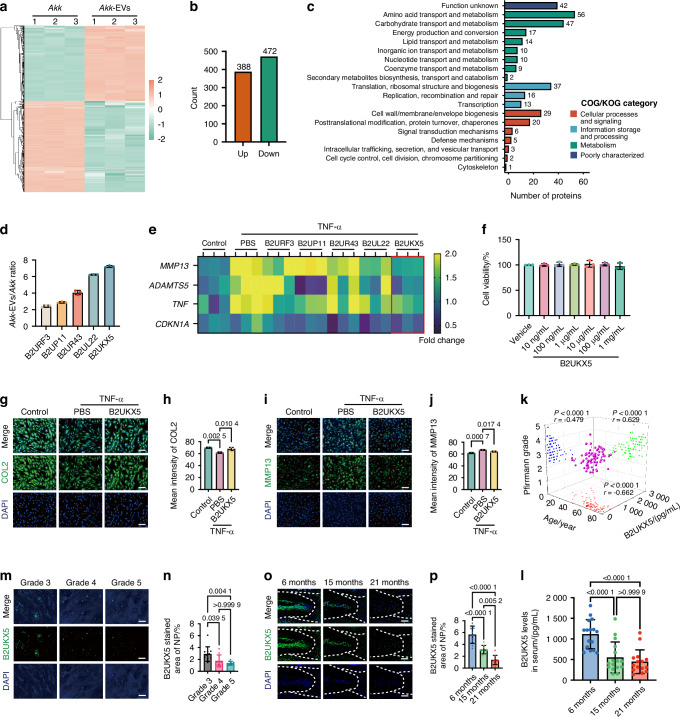
B2UKX5 as a potential functional protein in *Akk*-EVs against IVDD. Hierarchical clustering heatmap (**a**) and count (**b**) of differentially enriched proteins between *Akk* and *Akk*-EVs. **c** Functional annotation for the highly enriched 388 proteins using the COG database. **d** Relative enrichment levels of the five selected candidate proteins in *Akk*-EVs compared to *Akk*, displayed as *Akk*-EVs/*Akk* ratios. **e** Heatmap showing qPCR-based expression levels of *MMP13*, *ADAMTS5*, *TNF*, and *CDKN1A* in TNF-α-treated human NP cells following treatment with the five candidate proteins. *n* = 3 per group. **f** Cytotoxicity assessment of B2UKX5 in human NP cells using Cell Counting Kit-8 assay after 24 h treatment under indicated concentrations. Cell viability is expressed relative to vehicle control. *n* = 3 per group. **g** Representative IF staining of COL2 (green) in TNF-α-treated NP cells after B2UKX5 intervention; Nuclei are counterstained with DAPI (blue). Scale bar: 100 μm. **h** Quantification of COL2 mean fluorescence intensity in human NP cells. *n* = 3 per group. **i** Representative IF staining of MMP13 (green) in TNF-α-treated NP cells after B2UKX5 intervention; Nuclei are counterstained with DAPI (blue). Scale bar: 100 μm. **j** Quantification of MMP13 mean fluorescence intensity in human NP cells. *n* = 3 per group. **k** Correlation analysis between age, IVDD grading (Pfirrmann), and serum B2UKX5 levels in human subjects. *n* = 79. **l** Quantification of B2UKX5 levels in serum from mice of different age groups. *n* = 15–16 per group. **m** Representative IF staining of B2UKX5 (green) in human NP tissue from patients with varying degrees of IVDD; Nuclei are counterstained with DAPI (blue). Scale bar: 50 μm. **n** Quantification of B2UKX5-positive area (%) in human NP tissue; *n* = 9–11 per group. **o** Representative IF staining of B2UKX5 (green) in intervertebral disc sections from young versus aged mice; Nuclei are counterstained with DAPI (blue). Scale bar: 100 μm. **p** Quantification of B2UKX5-positive area (%) in mouse discs. *n* = 9–10 per group. Data are presented as mean ± SD. Statistical significance was assessed using one-way ANOVA followed by Bonferroni post hoc test, no significant differences were observed in panel **f**, and Pearson correlation was used for panel **k**

To identify candidate effector proteins responsible for *Akk*-EV-mediated protection, we performed quantitative proteomic analysis of *Akk* and *Akk*-EVs. Hierarchical clustering heatmaps revealed distinct protein expression profiles between *Akk* and *Akk*-EVs (Fig. 5a). Compared to *Akk*, *Akk*-EVs contained 388 proteins with significantly higher enrichment and 472 proteins with significantly lower enrichment (Fig. 5b). Functional annotation for the highly enriched 388 proteins using the clusters of orthologous groups (COG) database classified 315 of the highly enriched proteins into categories related to bacterial structure, defense mechanisms, vesicular transport, lipid transport, and carbohydrate transport (Fig. 5c), which are unlikely to play a direct role in IVDD protection. Among the remaining 73 proteins, we excluded highly conserved bacterial proteins and proteins localized in the bacterial periplasm, and then identified five candidate proteins, B2URF3, B2UP11, B2UR43, B2UL22, and B2UKX5, as potential mediators of IVDD protection (Fig. 5d).

To evaluate the therapeutic potential of these proteins, we utilized a TNF-α-induced IVDD model in human NP cells, which were identified by PCR detection of *KRT19* and *SOX9* (Fig. S12). Treatment of this cell model with the candidate proteins was followed by qRT-PCR analysis of *MMP13* and *ADAMTS5* (catabolic markers), *TNF* (inflammatory marker), and *CDKN1A* (senescence marker). The results revealed that B2UKX5 significantly reversed degeneration-associated gene expression (Fig. 5e). Importantly, Cell Counting Kit-8 assays confirmed that recombinant B2UKX5 exhibited no detectable cytotoxicity across all tested concentrations (Fig. 5f), supporting the safety of its use in subsequent cellular and in vivo functional analyses. IF staining further confirmed that B2UKX5 preserved COL2 expression while reducing MMP13 levels in TNF-α-treated NP cells, suggesting its role in delaying IVDD progression (Fig. 5g–j).

To validate the in vivo relevance of B2UKX5, we established a competitive ELISA using a self-prepared B2UKX5-specific antibody. Using the same cohort of 79 MRI-graded IVDD patients (aged 18–79 years; detailed patient information in Table S2), we quantified serum B2UKX5 levels, and the ELISA standard curve is shown in Fig. S3b. Correlation analysis in human serum samples demonstrated a significant negative association between serum B2UKX5 levels and IVDD severity (*P* < 0.000 1, *r* = −0.479). Furthermore, a positive correlation was observed between age and IVDD grade (*P* < 0.000 1, *r* = 0.629), and a negative correlation was identified between age and B2UKX5 levels (*P* < 0.000 1, *r* = −0.662; Fig. 5k). These findings suggest that reduced B2UKX5 levels are associated with more severe IVDD. A similar trend was observed in mouse serum samples, where B2UKX5 levels decreased with age (Fig. 5l). Furthermore, IF staining of human NP tissues across different IVDD grades (details in Table S2) and mouse disc tissues of varying ages revealed a progressive decline in B2UKX5 expression with increasing IVDD severity (Fig. 5m–p).

Collectively, these data suggest that among the five identified proteins, B2UKX5 may play a pivotal role in mediating the protective effects of *Akk*-EVs on IVDD.

### B2UKX5 supplementation alleviates IVDD

**Fig. 6:**
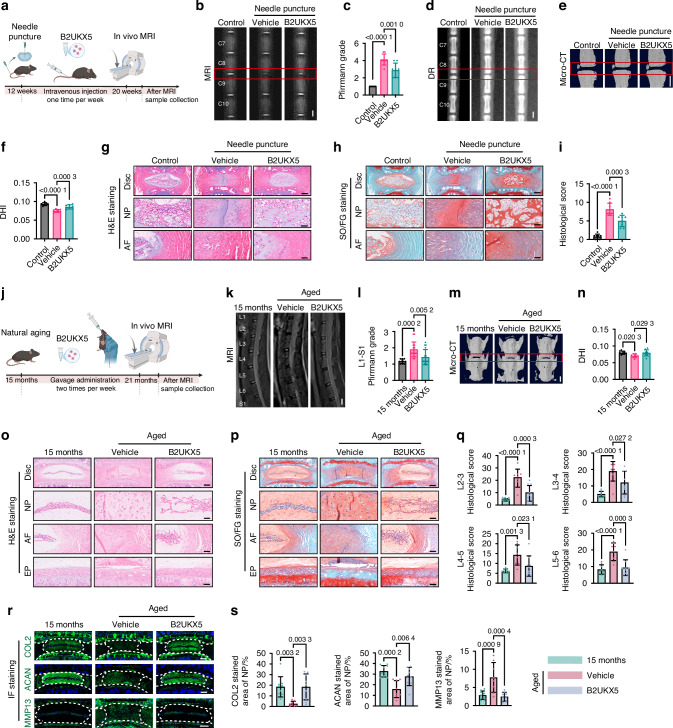
B2UKX5 Supplementation Alleviates IVDD. **a** Schematic of the experimental protocol for B2UKX5 treatment in tail needle puncture mice. **b** Representative T2-weighted MRI images of the C8-9 caudal spine. Scale bar: 1 mm. **c** Statistical analysis of Pfirrmann grading for the C8-9 caudal segment. *n* = 8 per group. Representative DR images (**d**) and micro-CT 3D reconstruction images (**e**) of the caudal spine. Scale bar: 1 mm. **f** DHI after 8 weeks of needle puncture at the C8-9 caudal segment. *n* = 8 per group. Representative H&E (**g**) and SO/FG (**h**) stained images showing the macroscopic morphology of the disc, NP, and AF in the C8-9 segment. Scale bars: 200 μm (Disc) or 50 μm (NP, AF). **i** Histological scores for the C8-9 segment. *n* = 8 per group. **j** Schematic of the experimental protocol for B2UKX5 treatment in naturally aging mice. **k** Representative T2-weighted MRI images of the lumbar spine in mice following B2UKX5 treatment. Scale bar: 1 mm. **l** Statistical analysis of the average Pfirrmann grade in the L1-S1 lumbar disc segments. *n* = 10–16 per group. **m** Representative micro-CT 3D reconstruction images of the L5-6 lumbar disc segments. Scale bar: 1 mm. **n** Statistical analysis of the mean DHI in the L2-6 lumbar disc segments. *n* = 8–9 per group. Representative images of H&E staining (**o**) and SO/FG staining (**p**) showing the macroscopic morphology of intervertebral discs (Disc), nucleus pulposus (NP), annulus fibrosus (AF), and endplate (EP) in the L4-5 lumbar disc segment. Scale bars: 200 μm (Disc) or 50 μm (NP, AF, EP). **q** Histological scores for each lumbar segment (L2-6). *n* = 8–9 per group. **r** Representative IF images showing expression of COL2, ACAN, and MMP13 in the L5-6 lumbar intervertebral disc segment. Scale bar: 200 μm. **s** Quantitative analysis of IF staining for COL2, ACAN, and MMP13. *n* = 8–9 per group. Data are presented as mean ± SD. Statistical significance was assessed by one-way ANOVA followed by Bonferroni post hoc test

Next, we explored whether systemic administration of B2UKX5 can attenuate IVDD. In the tail needle puncture model, punctured mice received vehicle or B2UKX5 once weekly for 8 weeks, with non-punctured mice as baseline controls (Fig. 6a). At the endpoint, T2-weighted MRI revealed markedly reduced degeneration in the B2UKX5-treated group, and DHI measurements from DR and micro-CT showed that B2UKX5 significantly maintained disc height compared with vehicle (Fig. 6b–f). Consistently, histological evaluation further demonstrated preserved NP cellularity, improved matrix organization, and fewer AF fissures in the B2UKX5 group (Fig. 6g–i).

We next evaluated orally administered B2UKX5 in the natural aging model using the same regimen and readouts established above (Fig. 6j). MRI scans showed preserved T2 signal intensity in B2UKX5-treated mice, reflected by lower mean Pfirrmann grades compared with vehicle controls (Fig. 6k, l). Consistently, micro-CT demonstrated maintenance of disc height, with higher DHI in the B2UKX5 group (Fig. 6m, n). Histology further indicated attenuation of age-associated structural disruption across lumbar levels (L2-6; Fig. 6o–q), and IF analysis supported preserved ECM homeostasis, with maintained COL2 and ACAN signals and reduced MMP13 (Fig. 6r, s).

Consistent with the findings from *Akk* and *Akk*-EVs, B2UKX5 did not produce detectable changes in endplate porosity or osteogenic/osteoclastic markers, suggesting no appreciable effects on endplate remodeling in aged mice (Fig. S13). Within the disc, B2UKX5 reduced the p16^Ink4a^ and TNF-α signals, whereas IL-6 remained unchanged (Fig. S14a, b). Systemic inflammatory cytokines and serum biochemistry were not altered (Fig. S14c, d). Major organs showed only age-related changes, with no evidence of additional B2UKX5-related toxicity (Fig. S14e).

Taken together, these data demonstrate that B2UKX5 is sufficient to reproduce key disc-protective effects of *Akk*-EVs in vivo. Across both acute injury and age-related degeneration models, B2UKX5 consistently delayed disc degeneration. Notably, long-term B2UKX5 administration did not induce systemic inflammation or organ toxicity.

### B2UKX5 induces significant transcriptomic regulation in the NP region

**Fig. 7:**
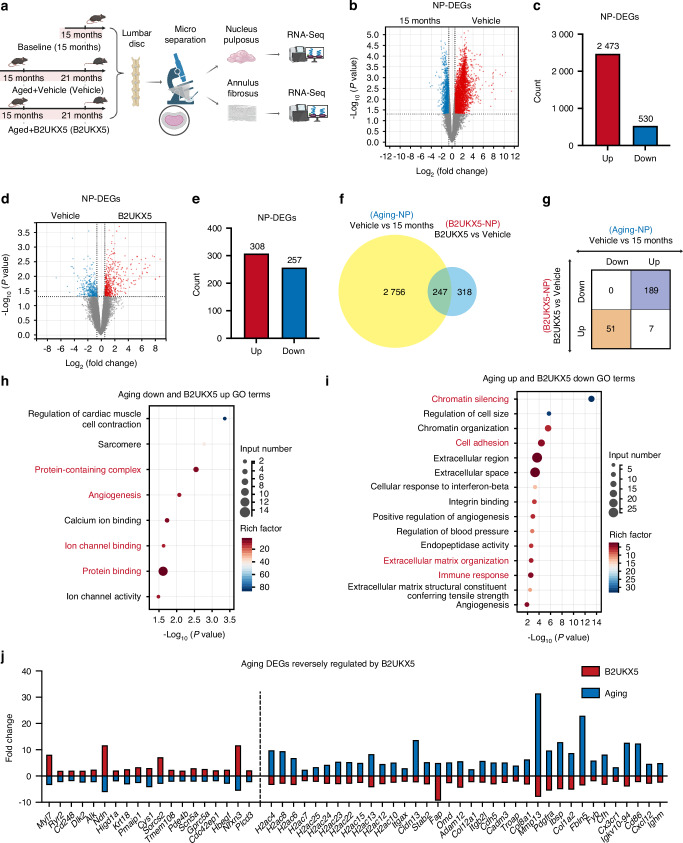
B2UKX5 induces significant transcriptomic regulation in the nucleus pulposus region. **a** Flowchart illustrating the experimental design for transcriptomic analysis in the natural aging model. Volcano plot of DEGs (**b**) and directional DEG counts (**c**) in NP tissue between the vehicle-treated and 15-month-old groups. Volcano plot of DEGs (**d**) and directional DEG counts (**e**) in NP tissue between the B2UKX5-treated and vehicle-treated groups. **f** Venn diagram illustrating the overlap of DEGs in B2UKX5-treated and aging-related datasets. **g** Number of genes inversely regulated by B2UKX5 treatment relative to aging. **h** GO analysis of DEGs downregulated in aging and upregulated by B2UKX5 treatment. **i** GO analysis of DEGs upregulated in aging and downregulated by B2UKX5 treatment. **j** List of representative DEGs with inverse regulation by B2UKX5 in the aging process

Having established the in vivo efficacy of B2UKX5, we next sought to define the disc-intrinsic molecular programs regulated by this protein under physiologically relevant degenerative conditions. Because age-related IVDD reflects the chronic, progressive nature of human disc degeneration and oral delivery represents the most translatable route of exposure, we performed transcriptome profiling of NP and AF tissues isolated from aged mice following long-term oral B2UKX5 supplementation. The isolated tissues were subjected to transcriptome sequencing to investigate gene expression alterations induced by B2UKX5 (Fig. 7a). Principal component analysis demonstrated distinct clustering of NP and AF tissues, confirming differential gene expression patterns between these two regions (Fig. S15a). NP-specific marker genes, including *Krt19*, *Slc2a1*, and *Car3*, exhibited high expression in NP samples, whereas AF-specific markers such as *Comp*, *Cilp*, and *Prg4* were highly expressed in AF tissues^46,47^ (Fig. S15b). These findings confirmed the accuracy of tissue microdissection and the robustness of transcriptome sequencing.

We first analyzed differential gene expression in NP tissues from vehicle-treated versus 15-month-old control groups. This comparison identified 3 003 differentially expressed genes (DEGs), with 2 473 upregulated and 530 downregulated in vehicle-treated NP tissues (Fig. 7b, c). Gene Ontology (GO) enrichment analysis revealed significant changes in immune response and inflammatory pathways, consistent with aging-related IVDD pathology (Fig. S15c).

To determine the impact of B2UKX5 on NP tissue, we compared gene expression profiles between B2UKX5-treated and vehicle-treated groups. A total of 565 DEGs were identified, including 308 upregulated and 257 downregulated genes in the B2UKX5-treated NP tissues (Fig. 7d, e). GO enrichment analysis indicated significant involvement of collagen biosynthesis, extracellular matrix organization, and chromatin silencing pathways, suggesting that B2UKX5 exerts its effects by modulating ECM homeostasis and epigenetic regulatory mechanisms (Fig. S15d). Additionally, genes associated with angiogenesis and immune response were significantly enriched (Fig. S15d), indicating broader regulatory functions of B2UKX5.

A Venn diagram analysis revealed 247 overlapping DEGs between aging-related and B2UKX5-treated groups. Of these, 51 genes were downregulated during aging but upregulated upon B2UKX5 treatment, while 189 genes exhibited the opposite trend (Fig. 7f, g). GO analysis of these genes highlighted several key functional categories: genes involved in protein complex stability (*Alk*, *Ndn*, *Higd1a*, *Krt18*), angiogenesis (*Hbegf*, *Plcd3*, *Nrxn3*), ion channel activity (*Myl7*, *Ryr2*, *Cd248*, *Dlk2*), and cell adhesion (*Pmaip1*, *Cys1*, *Sorcs2*, *Tmem108*, *Pde4b*, *Scn5a*, *Gorc5a*, *Cdc42ep1*; Fig. 7h, j). Among genes that were upregulated during aging and further reversed by B2UKX5 treatment, chromatin silencing genes (*H2ac4*, *H2ac8*, *H2ac7*, *H2ac6*, *H2ac24*, *H2ac25*, *H2ac22*, *H2ac23*, *H2ac15*, *H2ac13*, *H2ac12*, *H2ac10*), cell adhesion regulators (*Itgax*, *Cldn13*, *Stab2*, *Fap*, *Omd*, *Adam12*, *Col12a1*, *Itgb2l*, *Cdh5*, *Cadm3*, *Troap*), ECM-associated genes (*Col8a1*, *Mmp13*, *Pdgfra*, *Col1a2*, *Ibsp*, *Fbln5*), and immune response genes (*Fyb*, *Cfh*, *Cx3cr1*, *Igkv10-94*, *Cd86*, *Cxcl12*, *Ighm*) were prominently represented (Fig. 7i, j).

These results suggest that B2UKX5 regulates NP metabolism by modulating ECM composition, chromatin dynamics, and immune response, ultimately contributing to the attenuation of IVDD progression.

### B2UKX5 induces significant transcriptomic regulation in the AF region

**Fig. 8:**
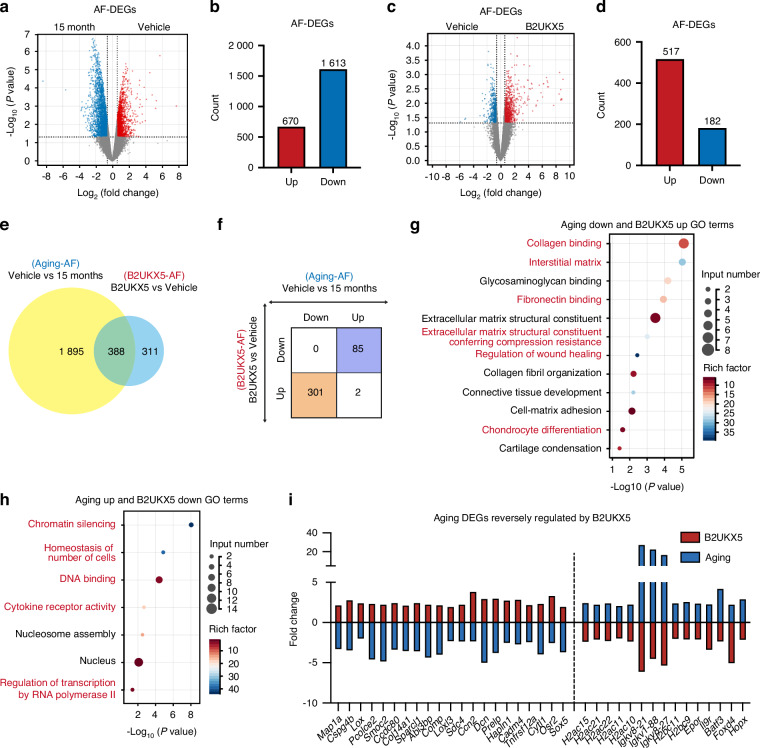
B2UKX5 induces significant transcriptomic regulation in the annulus fibrosus region. Volcano plot of DEGs (**a**) and directional DEG counts (**b**) in AF tissue between the vehicle-treated and 15-month-old groups. Volcano plot of DEGs (**c**) and directional DEG counts (**d**) in AF tissue between the B2UKX5-treated and vehicle-treated groups. **e** Venn diagram illustrating the overlap between DEGs in B2UKX5-treated and aging-related datasets. **f** Number of genes inversely regulated by B2UKX5 treatment relative to aging. **g** GO analysis of representative pathways for DEGs downregulated during aging and upregulated after B2UKX5 treatment. **h** GO analysis of representative pathways for DEGs upregulated during aging and downregulated after B2UKX5 treatment. **i** Representative DEGs in AF tissue that are inversely regulated by B2UKX5 treatment compared to the aging process

To further investigate the molecular mechanisms by which B2UKX5 mitigates IVDD, we also analyzed the transcriptomic profiles of AF tissues from vehicle-treated and 15-month-old mice. This analysis identified 2 283 DEGs, of which 670 were upregulated and 1613 were downregulated in vehicle-treated AF tissues (Fig. 8a, b). GO enrichment analysis revealed that these DEGs were primarily associated with ECM maintenance, collagen organization, cell adhesion, skeletal system development, and chondrogenesis, highlighting critical pathways affected by age-related degeneration (Fig. S15e).

To evaluate the transcriptional effects of B2UKX5, we performed RNA sequencing of AF tissues from B2UKX5-treated and vehicle-treated groups. This analysis identified 699 DEGs, with 517 genes significantly upregulated, and 182 genes significantly downregulated in the B2UKX5-treated group compared to controls (Fig. 8c, d). GO analysis of these DEGs revealed significant enrichment in pathways related to collagen biosynthesis, ECM integrity, fibronectin binding, cytokine response, calcium binding, and chromatin silencing, suggesting that B2UKX5 exerts regulatory effects on both ECM composition and gene transcription (Fig. S15f).

A Venn diagram comparison of DEGs from both aging and B2UKX5 treatment datasets identified 388 overlapping genes (Fig. 8e). Among these, 301 genes were downregulated during aging but upregulated following B2UKX5 treatment, whereas 85 genes were upregulated during aging and suppressed by B2UKX5 treatment (Fig. 8f). Functional categorization of these genes provided insight into key biological processes modulated by B2UKX5.

Genes downregulated during aging but restored by B2UKX5 treatment included *Map1a*, *Cspg4b*, *Lox*, *Pcolce2*, which are involved in collagen binding; interstitial matrix regulators such as *Smoc2*, Ccdc80, *Col14a1*, *Sparcl1*, and *Abi3bp*; fibronectin-binding proteins including *Comp*, *Loxl3*, *Sdc4*, and *Ccn2*; ECM stress-resistance genes such as *Dcn*, *Prelp*, and *Hapln1*; wound healing regulators *Cadm4* and *Tnfrsf12a*; and chondrocyte differentiation markers *Cytl1*, *Osr2*, and *Sox5* (Fig. 8g, i). Conversely, genes upregulated during aging and suppressed by B2UKX5 treatment included histone genes associated with chromatin silencing (*H2ac15*, *H2ac21*, *H2ac22*, *H2ac11*, *H2ac10*); genes involved in cell number homeostasis (*Igkv8-21*, *Igkv1-88*, *Igkv8-27*); DNA-binding regulators (*H2bc11*, *H2bc9*); cytokine receptor genes *Epor* and *Il9r*; and transcriptional regulators of RNA polymerase II, including *Batf3*, *Foxd4*, and Hopx (Fig. 8h, i).

These results suggest that B2UKX5 plays a critical role in maintaining AF homeostasis by promoting ECM integrity, regulating mesenchymal and collagen-related pathways, and modulating transcriptional and chromatin dynamics.

## Discussion

**Fig. 9:**
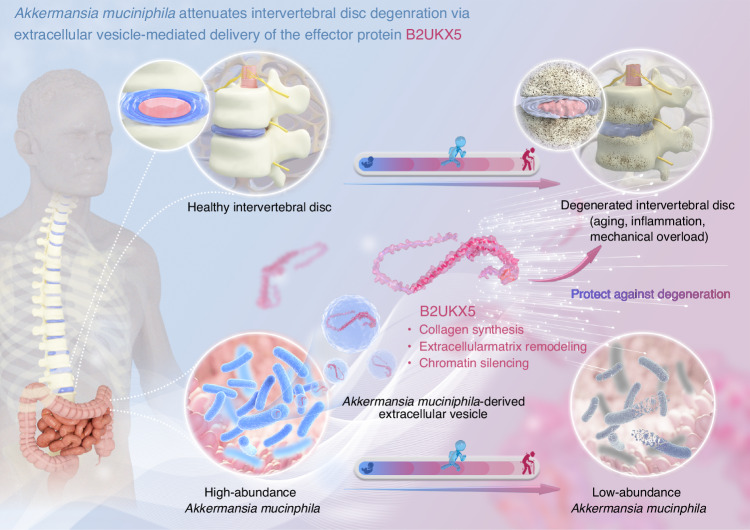
*Akkermansia muciniphila* attenuates intervertebral disc degeneration via extracellular vesicle-mediated delivery of the effector protein B2UKX5. *Akkermansia muciniphila* produces extracellular vesicles (*Akk*-EVs) enriched with the effector protein B2UKX5, which are transported to the intervertebral disc. B2UKX5 improves collagen synthesis, promotes extracellular matrix remodeling, and modulates chromatin silencing-related pathways in intervertebral disc cells, thereby attenuating intervertebral disc degeneration

The present study demonstrates a novel role for *Akk*, *Akk*-EVs, and its carried key protein B2UKX5 in modulating IVDD. Integrating MR analysis, a clinical fecal cohort, and in vivo intervention experiments with microbiota depletion to minimize confounding, we obtained convergent evidence that lower *Akk* abundance tracks with more severe IVDD and that *Akk* supplementation functionally mitigates disc degeneration. Mechanistically, GW4869-mediated inhibition of EVs secretion blocked *Akk*’s protection, while direct administration of *Akk*-EVs (oral or intravenous) reached disc tissues and recapitulated the protective phenotype. Finally, comparative proteomics and gain-of-function experiments identified B2UKX5 as an *Akk*-EV–enriched cargo sufficient to phenocopy *Akk*-EVs in vivo, accompanied by compartment-resolved transcriptomic programs consistent with restored collagen/ECM homeostasis and chromatin regulation in NP and AF tissues. Collectively, these results delineate an EV-dependent gut–disc communication axis mediated by *Akk*-EVs and B2UKX5, providing a mechanistic framework for the development of *Akk*-derived postbiotics for IVDD (Fig. 9).

IVDD continues to pose significant clinical challenges due to the absence of effective prevention or treatment options. While there has been growing interest in the relationship between the gut microbiota and IVDD,^15–19^ most studies have yet to identify specific microbial species or validate their functional roles in vivo. Our study addresses this gap by identifying *Akk* as a key bacterium capable of influencing IVDD progression. Using Mendelian randomization, human stool and tissue analyses, and functional assessment in multiple mouse models, we show that *Akk* protection depends on its secretion of EVs enriched in the protein B2UKX5. Notably, our findings identify B2UKX5 as a previously uncharacterized protein whose biological function has not been reported, and we provide the first evidence that it mitigates IVDD, offering new insight into its underlying molecular mechanisms.

Age is one of the strongest and most unavoidable risk factors for IVDD, underscoring the importance of evaluating preventive strategies under physiologically relevant conditions. Our use of a natural aging model therefore enabled assessment of long-term protective effects within a chronic, low-grade degenerative micro-environment characteristic of human IVDD. Because disc degeneration arises from distinct etiological drivers beyond aging, we further incorporated a tail-needle puncture model, which recapitulates acute inflammatory injury, and a bipedal standing model, which mimics sustained mechanical overload. These models collectively span the major pathogenic pathways implicated in human IVDD: senescence-related matrix exhaustion, inflammation-induced catabolism, and mechanical stress-driven structural failure. Importantly, the protective effects of *Akk* and its derivatives were highly consistent across these divergent pathological contexts. This cross-model robustness suggests that the *Akk*-EVs-B2UKX5 axis acts on fundamental biological processes shared among different forms of disc degeneration rather than on model-specific features. Such consistency substantially strengthens the translational relevance of our findings, as it indicates that *Akk*-based interventions may retain efficacy across heterogeneous clinical presentations of IVDD.

Notably, the disc-protective effects of *Akk*, *Akk*-EVs, and B2UKX5 were independent of alterations in cartilaginous endplate bone remodeling. In the natural aging model, micro-CT and immunohistochemical analyses revealed no significant changes in endplate Porosity or osteoblast/osteoclast activity following treatment (Figs. S9 and S13). This lack of effect may reflect the pathological “high-turnover” state characteristic of aged/degenerative endplates, where enhanced osteoclast-mediated resorption and disorganized osteoblast activity lead to sclerotic yet structurally compromised bone.^48^ In this context, the osteoprotective actions of *Akk*-EVs observed in healthier bone models may be masked, further underscoring that the benefits in IVDD arise primarily from direct actions on disc cellular function and extracellular matrix homeostasis rather than secondary effects via the disc–bone interface.

Obesity and type 2 diabetes are prevalent predisposing factors for IVDD,^49–52^ with *Akk* populations often diminished in individuals affected by these conditions.^53,54^ Previous studies have indicated that *Akk* supplementation can alleviate metabolic disorders by enhancing intestinal barrier function, reducing systemic inflammation, and modulating energy metabolism^53,55,56^ (e.g., improving insulin sensitivity and reducing fat accumulation). By ameliorating these underlying conditions, *Akk* supplementation could potentially reduce the systemic inflammation and metabolic dysregulation that contribute to IVDD progression. However, *Akk* supplementation as a live bacterial formulation poses several challenges.^57^ While clinical trials have examined the effects of *Akk* on obesity and diabetes (e.g., NCT02637115), it has not yet received approval as a therapeutic agent. Safety concerns, particularly in immunocompromised populations, and stability issues, given the strict anaerobic nature and costly production and storage, impede its broader application.^58,59^ Moreover, the use of live bacteria as pharmaceuticals necessitates rigorous regulatory oversight. To address these challenges, *Akk*-EVs and their functional proteins, such as Amuc_1100^24^ and B2UKX5, are being explored as “postbiotics” to circumvent the complications associated with live bacterial therapies, providing a promising avenue for therapeutic development.

In our study, we employed microdissection techniques to isolate NP and AF tissues, overcoming the challenge of sequencing low-abundance and easily degraded intervertebral disc tissues. This approach enabled us to obtain high-resolution transcriptomic data, which revealed that B2UKX5 plays a key role in regulating molecular pathways associated with IVDD attenuation. Specifically, B2UKX5 influenced processes such as chromatin silencing and ECM remodeling in both NP and AF tissues. Notably, B2UKX5 itself represents a previously uncharacterized EV-associated effector. According to the UniProt database (accession B2UKX5), it is a 31.5-kD protein encoded by the *Akk* gene *Amuc_1426* and is annotated as a hypothetical protein with no known structural domains or prior biological characterization. Our study is the first to identify B2UKX5 as a functional bacterial effector that can attenuate intervertebral disc degeneration in mice. We further demonstrate that B2UKX5 regulates genes involved in epigenetic modulation within both NP and AF tissues—an area in which the regulatory roles of bacterial proteins have not previously been explored. These findings suggest that bacterial proteins such as B2UKX5 may modulate the epigenetic landscape of disc cells, providing new insights into the molecular pathways that drive IVDD progression.

While this study provides compelling evidence for the role of *Akk*, *Akk*-EVs, and B2UKX5 in modulating IVDD, several limitations should be acknowledged. First, although our data show that orally administered *Akk*-EVs can be absorbed through the intestine, enter the systemic circulation, and become detectable in disc tissues, the precise trafficking route from gut to disc remains to be defined. Based on the spatial distribution of *Akk*-EV signals (enriched in vertebral bone/endplate regions, the outer annulus fibrosus, and the nucleus pulposus, with little signal in the inner annulus), we speculate that circulating *Akk*-EVs may access the disc primarily via endplate-associated blood supply and subsequently diffuse toward the nucleus pulposus; this inference is included as a limitation and will be addressed by future in vivo tracing studies. Second, we also acknowledge that we did not directly test necessity by generating a B2UKX5-deficient *Akk* strain (e.g., targeted deletion of *Amuc_1426*) in the current study, as we have not yet been able to establish a genetically stable *Amuc_1426*-deficient *Akk* strain suitable for in vivo experiments. Hu et al. generated an ldhA-deficient *E. coli* strain using CRISPR-based editing and homologous recombination,^60^ and we plan to adapt such approaches to construct an *Amuc_1426*-deficient *Akk* strain, verify EVs cargo depletion, and then evaluate its in vivo phenotype, ideally with genetic complementation rescue. Third, although a range of in vivo models have been employed to assess different aspects of IVDD, the translation of these results to large animal and human clinical settings remains uncertain. Fourth, although the study demonstrated the potential protective effects of *Akk* and its derivatives in multiple animal models, the exact mechanisms by which these entities exert their effects in vivo remain partially understood. The study of the pathomechanisms of IVDD is significantly limited by the highly heterogeneous nature of intervertebral disc tissue.^61–63^ The intricacy of intervertebral disc cells and the ambiguity surrounding nucleus pulposus stem cells complicate the analysis of the specific cells and their mechanisms that are predominantly responsible for the therapeutic effect.^64,65^ In addition, the small tissue volume of rodent discs imposes practical constraints on high-resolution sequencing and other deep mechanistic profiling, further limiting cell-resolved mechanistic dissection in vivo. Nevertheless, although transcriptomic analyses provided clues regarding pathways potentially regulated by B2UKX5, further mechanistic studies are required to define its receptors, downstream signaling cascades, and cell-type–specific actions within the disc. Elucidating these mechanisms of transport and signaling will be essential for advancing *Akk*-based therapeutics toward clinical translation.

In conclusion, our study provides a comprehensive framework linking *Akk*, its EVs, and EV-carried proteins to the maintenance of disc homeostasis. Rather than focusing on a single molecular event, the data collectively illustrate that microbial derivatives can engage multiple disc-protective pathways and act across diverse degenerative contexts. The identification of B2UKX5 as one EV-borne effector highlights only one component of a broader regulatory repertoire, underscoring the potential richness of EV cargo yet to be explored. These findings suggest that microbial molecules, independent of live bacterial administration, may be leveraged as stable and controllable agents to modulate disc biology. Viewed collectively, these insights extend the concept of microbe–host communication to the intervertebral disc and provide a foundation for developing next-generation, postbiotic strategies to delay or prevent IVDD.

## Materials and methods

### MR analysis

MR was conducted using genome-wide association studies. IVDD data were sourced from the FinnGen consortium (41 669 cases, 294 770 controls),^66^ while *Akk* abundance data came from a population-based cohort of 5 959 individuals with genotypic and metagenomic records.^67^

MR analysis is based on three core assumptions^68^: (i) IVs must be strongly associated with the exposure; (ii) the IVs should not be associated with confounders; and (iii) the genetic variants must influence the outcome solely through the exposure. To ensure robustness, we selected IVs with a genome-wide significance threshold of *P* < 5 × 10^−5^.^69^ Linkage disequilibrium pruning was applied (*r*^2^ < 0.001, window size < 10 Mb) to retain independent IVs. Additionally, IVs exhibiting direct associations with the outcome were excluded to prevent bias due to horizontal pleiotropy. To further eliminate confounding effects, IVs associated with common risk factors for IVDD, including obesity, alcohol consumption, and smoking behavior, were excluded.

A two-sample MR framework was implemented using seven complementary analytical methods: random-effects IVW, weighted median, simple mode, weighted mode, penalized weighted median, MR-Egger, and maximum likelihood estimation. IVW was designated as the primary method, as it provides the highest statistical power under the assumption that all IVs are valid and free from pleiotropy.^70,71^ Cochran’s Q test and funnel plot analysis were used to assess heterogeneity among IVs.^72^ The presence of horizontal pleiotropy was evaluated using the MR-Egger intercept test,^73,74^ while a leave-one-out sensitivity analysis was conducted to determine whether individual SNPs disproportionately influenced the results, thereby validating compliance with the second and third MR assumptions.^75^

### Human samples

Ethical approval for human sample collection and analysis was obtained from the Ethics Committee of the Third Xiangya Hospital, Central South University (Approval No. Fast 24932 and 25804). Two independent clinical cohorts were included in this study.

#### Cohort 1: Fecal samples for *Akk* abundance analysis

A total of 62 inpatients from the Department of Spinal Surgery, Third Xiangya Hospital, provided fecal samples. The cohort included 34 males and 28 females. Disc degeneration severity was assessed using the MRI-based Pfirrmann grading system. Fecal DNA was extracted, and the relative abundance of *Akk* was quantified by qRT-PCR, followed by correlation analysis with IVDD severity.

#### Cohort 2: Serum and intervertebral disc tissue samples for *Akk*-EVs and B2UKX5 analysis

An additional 92 inpatients were enrolled, including 40 females and 52 males. IVDD severity was evaluated using the MRI-based Pfirrmann grading system. Serum samples from 79 patients were used to assess the association between Pfirrmann grade and *Akk*-EVs or B2UKX5 levels using competitive ELISA. NP tissues from 29 patients were collected intraoperatively for immunofluorescence staining, enabling evaluation of *Akk*-EVs and B2UKX5 abundance within disc tissue.

Participants were excluded if they met any of the following conditions:

1. Use of systemic antibiotics, probiotics, corticosteroids, or immunosuppressive agents within the past 3 months, due to their known impact on gut microbiota composition;
2. Acute traumatic disc injury, spinal fracture, or prior lumbar spine surgery;
3. History of spinal infection, spinal tumor, congenital spinal deformity, or inflammatory spondyloarthropathy (e.g., ankylosing spondylitis);
4. Active smoking or a smoking history ≥10 pack-years;
5. Occupational mechanical overload, including heavy manual labor or long-term repetitive lumbar flexion;
6. Severe obesity (BMI ≥ 35 kg/m²);
7. Severe systemic conditions that affect metabolism, inflammation, or gut microbiota, including uncontrolled diabetes, chronic kidney or liver disease, autoimmune disorders, or other chronic inflammatory diseases.

### Animal models

Abx microbiota depletion model: Freshly prepared antibiotics (1 μg/mL bacitracin, 170 μg/mL gentamicin, 125 μg/mL ciprofloxacin, 100 μg/mL neomycin, 100 U/mL penicillin, 100 μg/mL metronidazole, 100 μg/mL ceftazidime, 50 μg/mL streptomycin, and 50 μg/mL vancomycin; Solarbio) were added to drinking water and replaced twice per week. After 4 weeks of treatment, mice were returned to normal water.^36^ Gut microbiota depletion was confirmed by fecal supernatant plating and reduced fecal DNA yield, after which IVDD was induced using the needle-puncture procedure described above.

In the tail needle–puncture model, IVDD was induced in 12-week-old male C57BL/6 J mice by inserting a 31-G needle into the C8–9 caudal disc to a depth of 1.5 mm. For all 8-week intervention studies (Abx+*Akk*, Abx+*E. coli*, *Akk*, *Akk*-GW4869, *Akk*-EVs, *E. coli*-EVs, and B2UKX5), puncture was performed by rotating the needle 180° and maintaining the position for 30 seconds. For the 12-week intervention study (*Akk* and *Akk*-EVs in Fig. 4), an alternative puncture procedure was used, in which the needle was inserted and then slowly rotated axially for 30 seconds.

For bacterial supplementation in Abx-depleted mice, *Akk* or *E. coli* was administered by oral gavage at 1.5 × 10^8^ CFU per dose, three times per week. *Akk* and *Akk*-GW4869 preparations under normal microbiota conditions were administered using the same regimen (1.5 × 10^8^ CFU per dose, three times per week). For EVs supplementation, mice received *Akk*-EVs or *E. coli*-EVs by oral gavage at 100 μg per dose, three times per week, or by tail-vein injection at 30 μg per dose, once per week. For protein supplementation, B2UKX5 was administered by tail-vein injection at 30 μg per dose, once per week.

Natural aging model: 15-month-old male mice were randomly assigned to the following groups: Vehicle (PBS), *Akk* (1.5 × 10^8^ CFU), *Akk*-EVs (100 µg), and B2UKX5 (50 µg). All treatments were administered via oral gavage twice weekly for 6 months. At 21 months, mice underwent lumbar MRI under anesthesia, and blood samples were collected for serum analysis. Mice were then euthanized to harvest lumbar (L2-6) and other organ tissues for further evaluation.

Bipedal standing model: 8-week-old male mice were randomly divided into Control, Model (bipedal standing), *Akk* (3.0 × 10^8^ CFU), and *Akk*-EVs (100 µg) groups. Treatments were administered via oral gavage twice weekly for 12 weeks. Mice in the model and treatment groups were placed in a confined space with shallow water (5 mm) to induce a bipedal stance for 8 h daily, 7 days per week.^44^ After 12 weeks, mice were sacrificed, and lumbar (L2-6) tissues were collected for further analysis.

All procedures were approved by the Experimental Animal Ethics Committee of Xiangya Hospital, Central South University (Ethical approval No. 202404065).

### Stool culture measurement

To assess the reduction of gut microbiota after antibiotic treatment, fecal samples were collected from vehicle-treated and Abx-treated mice after 4 weeks of antibiotic exposure. Each sample was weighed and homogenized in 1 mL of PBS. The homogenate was briefly centrifuged, and the resulting supernatant was diluted 1:10. Equal volumes of diluted fecal supernatants from both groups were plated onto LB agar and incubated at 37°C for 48 h, after which CFUs were recorded.

### Bacterial culture and EV isolation

*Akk* (BNCC341917, BNCC) was cultured anaerobically at 37 °C in brain-heart infusion (BHI) broth (HB8297-5, Hope Bio-Technology) supplemented with 0.05% L-cysteine-HCl (C6183, Macklin). The bacterial concentration was determined via optical density measurement at 600 nm and correlated with CFU based on a standard curve. *Akk*-EVs were isolated by ultracentrifugation following established protocols.^30^

### Inhibition of extracellular vesicle secretion

*Akk* from the same starter culture was split into two groups and grown in BHI medium containing either vehicle (DMSO) or GW4869 (S7609, Selleck), generating bacteria with normal EVs secretion or inhibited EVs secretion, respectively. After 4 days of anaerobic culture, bacterial cells were harvested for subsequent gavage administration. To verify the efficiency of EVs suppression, culture supernatants were collected, and EVs were isolated and quantified by NTA.

### *Akk*-EVs tracing

*Akk*-EVs were labeled with the lipophilic dye DiR iodide (40757ES25, Yeasen). Eight-week-old mice (*n* = 3 per group) received intravenous injections of 100 µg of DiR-labeled *Akk*-EVs. 24 h post-injection, mice were euthanized, and intervertebral discs (L2-6) were isolated. Fluorescence intensity was measured using the IVIS Spectrum imaging system (2019026844, PerkinElmer). Quantitative analysis was performed by averaging fluorescence intensity across the L2-6 segments for each mouse.

### Proteomic analysis

Label-free quantitative proteomic analysis of *Akk* and *Akk*-EVs (*n* = 3 per group) was conducted by Hangzhou Jingjie Biotechnology Co., Ltd. Differentially enriched proteins were identified based on a fold change ≥ 2.0 or ≤0.5 and a *P* value < 0.05. GO analysis was performed to determine functional enrichment of identified proteins.

### Preparation of recombinant proteins and specific antibodies

Recombinant proteins B2UKX5, B2UL22, B2UP11, B2UR43, and B2URF3 were produced by Nanjing GenScript Biotechnology Co., Ltd. Proteins were purified to ≥ 90% purity, as confirmed by SDS-PAGE under reducing conditions. Endotoxin levels were maintained at ≤0.2 EU/mg.

Polyclonal antibodies against *Akk*-EVs and B2UKX5 were generated in rabbits using established immunization protocols and validated by Wuhan FriendBio Technology Co., Ltd. (*Akk*-EVs) and Nanjing GenScript Biotechnology Co., Ltd. (B2UKX5), respectively.

### Indirect competitive ELISA

*Akk*-EVs (10 µg/mL) or B2UKX5 (0.4 µg/mL) were coated onto uncoated ELISA plates (423510, BioLegend) and incubated overnight at 4 °C. Plates were then blocked with ELISA Assay Diluent (421203, BioLegend) for 1 h at 37 °C. Primary antibodies against *Akk*-EVs (1:2 000 dilution) or B2UKX5 (1:32 000 dilution) were applied and incubated for 1 h at 37 °C. After washing, HRP-conjugated goat anti-rabbit secondary antibody (G1213-100UL, Servicebio; 1:10 000 dilution for *Akk*-EVs, 1:20 000 dilution for B2UKX5) was applied for 30 minutes at 37 °C. Detection was performed using TMB High Sensitivity Substrate Solution (421501, BioLegend) for 3 minutes, followed by the Stop Solution for TMB Substrate (423001, BioLegend). Absorbance was measured at 450 nm within 15 minutes. A standard curve was established to calculate the concentration of *Akk*-EVs or B2UKX5 in samples (Fig. S3a, b), with raw data presented in Table S2.

### Imaging analysis of IVDD

MRI: For the natural aging model, MRI was performed on anesthetized mice to evaluate IVDD using a Siemens Prisma 3.0 T MRI scanner. Imaging parameters included a repetition time of 2 500 ms, an echo time of 65 ms, and a slice thickness of 0.8 mm. The L1-S1 intervertebral discs were assessed based on the Pfirrmann classification (grades 1 to 5).^76^

For the tail puncture model, MRI scans were performed on anesthetized mice using a Philips Ingenia 3.0 T MRI scanner with a repetition time of 3 000 ms, an echo time of 100 ms, and a slice thickness of 1.2 mm. The severity of intervertebral disc degeneration at needle-punctured segments was evaluated using the Pfirrmann classification.

DR: For the tail puncture model, DR was performed on anesthetized mice to assess the caudal vertebrae using a Philips DuraDiagnost F30 digital X-ray system. Imaging parameters included an exposure voltage of 40 kV and a current of 10 mAs.

micro-CT: The lumbar and caudal vertebrae were subjected to micro-CT analysis following fixation in 4% paraformaldehyde. Scanning was performed using a vivaCT80 scanner (SCANCO Medical AG) at a voltage of 50 kV, a current of 400 μA, and a resolution of 18 μm per pixel. The raw data were acquired using the micro-CT V6.1 software. IVDD was assessed by calculating the DHI using CT Analyser 1.11.0.0, µCTVol 2.2.0.0, and Dataviewer 1.4.3. The DHI was calculated using the formula: DHI = 2 × (DH1 + DH2 + DH3)/(A1 + A2 + A3 + B1 + B2 + B3), where A and B represent the lengths of the adjacent cranial and caudal vertebral bodies, respectively, and DH denotes intervertebral disc height at three measurement points per disc to ensure accuracy.^77–79^ The full thickness of the cranial endplates from L2-6 lumbar discs was defined as the region of interest (ROI), and endplate porosity was quantified using CT Analyser (v1.11.0.0). Porosity was expressed as the percentage of void volume relative to the total endplate volume within the defined ROI.

### Histological and IF staining

Following human NP collection, samples were fixed in 4% paraformaldehyde for 48 h, dehydrated, embedded in paraffin, and sectioned into 3 μm slices. H&E staining was performed for histological evaluation. For mouse intervertebral disc analysis, lumbar (L2-6) and caudal (C8-9) discs were similarly fixed, decalcified in 20% EDTA, embedded, and sectioned. Sections were stained with H&E and SO/FG. Lumbar disc degeneration was graded using a standardized histopathological scoring system for mouse IVDD models,^80^ while caudal disc degeneration was assessed using a model-specific scoring system.^81^ For gut tissue analysis, mouse gut segments were collected, fixed, and processed for paraffin embedding and sectioning using the same protocol as described above. The resulting sections were subsequently used for immunofluorescence staining.

For OCN staining, sections were incubated with anti-OCN antibody (1:200; GB11233, Servicebio) at 4 °C overnight, followed by an HRP-conjugated goat anti-rabbit secondary antibody (1:200; G1213, Servicebio) and DAB chromogenic detection (ZLI-9018, ZSGB-BIO). TRAP staining was performed using a commercial kit (G1050-50T, Servicebio) according to the manufacturer’s instructions. OCN-positive and TRAP-positive cells were quantified within the defined endplate region of each disc segment.

For IF staining, paraffin-embedded sections were deparaffinized with xylene, rehydrated through graded ethanol, and subjected to antigen retrieval using 0.5 mmol/L EDTA buffer (pH 9.0). Sections were blocked with 10% goat serum before incubation with primary antibodies: COL2 (1:300; GB11021-100, Servicebio), ACAN (1:300; GB11373-100, Servicebio), MMP13 (1:300; GB11247-100, Servicebio), p16^Ink4a^ (1:200; GB111143-100, Servicebio), IL-6 (1:300; GB11117-100, Servicebio), *Akk*-EVs-specific antibody (1:50, custom-made, produced by Wuhan FriendBio Technology Co) and B2UKX5-specific antibody (1:50, custom-made, produced by Nanjing GenScript Biotechnology Co). After washing, sections were incubated with Alexa Fluor 488-conjugated goat anti-rabbit secondary antibody (1:400; 111-545-144, Jackson). Coverslips were mounted with a DAPI-containing antifade medium (GTX30920, GeneTex). Fluorescent images were acquired using a Zeiss ApoTome fluorescence microscope. Quantitative analysis was performed using ImageJ software, applying a thresholding function to remove background noise and measure the stained area within a defined ROI.

### Serum multiplex cytokine analysis

Serum cytokines were measured using a bead-based multiplex flow cytometry assay (74109, LEGENDplex^TM^ Multi-Analyte Flow Assay kits, BioLegend) following the manufacturer’s instructions. Briefly, serum samples were incubated with cytokine-specific capture beads and detection antibodies, followed by PE-conjugated streptavidin labeling. Samples were acquired on a flow cytometer, and cytokine concentrations were calculated from standard curves using the LEGENDplex™ Data Analysis Software.

### Cell culture and treatment

Human NP immortalized cells (icell-0028a, iCell Bioscience), established using an SV40 adenoviral vector,^82^ were cultured in a humidified incubator at 37 °C with 5% CO_2_. Cells were maintained in high-glucose DMEM (C3110-0500, Viva Cell) supplemented with 10% fetal bovine serum (A5669701, Gibco) and 1% penicillin-streptomycin (P1400, Solarbio). The culture medium was refreshed every three days, and cells from passages 5 to 10 were used for experiments.

Human NP immortalized cells were cultured under standard conditions and divided into the following groups: vehicle (control) group, TNF-α group, and TNF-α plus candidate protein groups. TNF-α (AF-200-01B, PeproTech) was applied at 20 ng/mL to induce degeneration, while each candidate protein was administered at 2 μg/mL. After a 24-h incubation, cells were collected for qRT-PCR analysis to evaluate gene expression changes associated with IVDD. Based on the qRT-PCR results, one candidate protein was selected for further validation in an additional experiment using immunofluorescence analysis to assess protein expression and cellular localization.

### qRT-PCR

Genomic DNA from fecal samples was extracted using the TIANamp Stool DNA Kit (DP328, Tiangen) and used for PCR-based quantification of *Akk* abundance. Total RNA from cells was isolated using the Ultrapure RNA Kit (CW0581M, CWBIO), and complementary DNA was synthesized with the Evo M-MLV RT Premix Kit (AG11728, Accurate Biology). qRT-PCR was performed using 2× SYBR Green qPCR Master Mix (Q111-02, Vazyme) on an FTC-3000 real-time PCR system. Primer sequences used for qRT-PCR are provided in Table S3.

### Cell IF staining

For IF, cells were seeded on glass coverslips and fixed with 4% paraformaldehyde for 30 minutes at room temperature. Following fixation, cells were blocked with 4% bovine serum albumin for 20 minutes and incubated with primary antibodies: COL2 (1:300; 28459-1-AP, Proteintech) and MMP13 (1:300; 18165-1-AP, Proteintech). After washing, cells were incubated with Alexa Fluor 488-conjugated goat anti-rabbit secondary antibody (1:400; 111-545-144, Jackson). Coverslips were mounted using a DAPI-containing antifade medium. Fluorescent images were acquired using a Zeiss ApoTome fluorescence microscope. ImageJ software was used for quantitative analysis, applying a threshold function to remove background noise and measure the stained area.

### RNA extraction, library preparation, and sequencing

Intervertebral discs from five lumbar and caudal segments were collected from each mouse (*n* = 3 per group). NP and AF tissues were meticulously dissected in ice-cold PBS under a microscope, following established protocols.^83^

Transcriptomics sequencing and data analysis were performed by Wuhan SeqHealth Technology Co., Ltd. Briefly, total RNA was extracted using TRIzol reagent (15596026, Invitrogen), with genomic DNA removed via DNase I treatment. RNA purity and integrity were confirmed by NanoDrop spectrophotometer (Thermo Fisher Scientific) and agarose gel electrophoresis. Samples with RIN ≥ 4 (Qsep100 system) underwent rRNA depletion using the Ribo-off™ rRNA Depletion Kit (Human/Mouse/Rat) (Illumina, Cat. No. MRZG12324). Strand-specific RNA libraries were prepared with the KC-Digital™ Stranded mRNA Library Prep Kit for Illumina® (Wuhan Seqhealth Co., Ltd., Cat. No. DR08502), incorporating 8-base UMIs to reduce bias. Strand-specific RNA libraries were prepared using the KC-Digital Stranded mRNA Library Prep Kit for Illumina (DR08502, Wuhan Seqhealth Co., Ltd.). Library fragments (200-500 bp) were selected and quantified before paired-end 150 bp (PE150) sequencing on a DNBSEQ-T7 platform (MGI Tech Co., Ltd.).

Raw sequencing reads were quality-filtered using Trimmomatic (v0.36) to remove low-quality sequences and adapter contamination. Deduplicated reads were mapped to the mouse reference genome using STAR (v2.5.3a) with default parameters. Gene expression levels were quantified using featureCounts (Subread v1.5.1) and normalized as RPKM. Differential expression analysis was conducted using edgeR (v3.12.1), with significance thresholds of *P* < 0.05 and fold change ≥ 2. GO enrichment analysis of differentially expressed genes was performed using KOBAS (v2.1.1), with a statistical significance cutoff of *P* < 0.05.

### Statistical analysis

Data are presented as mean ± SD and analyzed using GraphPad Prism 10. Pearson correlation analysis was performed to evaluate associations among three variables. One-way ANOVA was applied for comparisons among multiple groups, followed by Bonferroni post hoc test. Student’s *t*-test was used for pairwise comparisons. A *P* value < 0.05 was considered statistically significant.

## Data and materials availability

All data supporting the findings of this study are available within the article and its Supplementary Information or from public repositories. The raw transcriptomic data have been deposited in the NCBI Sequence Read Archive (SRA) under BioProject accession number PRJNA1231219. The mass spectrometry proteomics data are available in the PRIDE Archive under accession number PXD062069.

## Supplementary information

Supplementary information
