# Supplementary material for: Akkermansia muciniphila attenuates intervertebral disc degeneration via extracellular vesicle-mediated delivery of the effector protein B2UKX5
Source: Bone Res. 2026 May 20;14:56. doi: 10.1038/s41413-026-00541-5 (PMC13190703; doi:10.1038/s41413-026-00541-5)
Supplement: Supplementary file 1 — Supplementary information [file 41413_2026_541_MOESM1_ESM.docx]

**Supplementary materials**

***Akkermansia muciniphila* Attenuates Intervertebral Disc Degeneration via Extracellular Vesicle-Mediated Delivery of the Effector Protein B2UKX5**

Zhe Guan^1,2^, Xiaoxue Li^3^, Yixiao Chen^1,2^, Sheng Zhu^1,2^, Jie Wen^4^, Hongliang Zhou^1,2^, Chunyuan Chen^1,2^, Jianghua Liu^5^, Guoqiang Zhu^1,2^, Zhilin Pang^1,2^, Yiwei Liu^1,2^, Ling Jin^1,2^, Shiyu Zeng^5^, Yi Luo^1,2^, Xiaoxiao Gong^6^, Yu Yang^1,2^, Ya Chen^1,2^, Yang Wu^1,2^, Meidan Wan^7^, Hao Yin^1,2^, Yong Zhou^8^*, Zhenxing Wang^1,2,9^*, Hui Xie^1,2,9^*

**Affiliations**

^1^Department of Orthopedics, Movement System Injury and Repair Research Center, Xiangya Hospital, Central South University, Changsha, Hunan 410008, China

^2^Hunan Key Laboratory of Angmedicine, Changsha, Hunan 410008, China

^3^Department of Gastroenterology, The First Affiliated Hospital of Harbin Medical University, Harbin, Heilongjiang 150001, China

^4^Department of Neurosurgery, Xiangya Hospital, Central South University, Changsha, Hunan 410008, China

^5^The First Affiliated Hospital of University of South China, Hengyang, Hunan 421000, China

^6^Department of Respiratory Medicine, National Key Clinical Specialty, Branch of National Clinical Research Center for Respiratory Disease, Xiangya Hospital, Central South University, Changsha, Hunan 410008, China

^7^Department of Neurology, Xiangya Hospital, Central South University, Changsha, Hunan 410008, China

^8^Third Xiangya Hospital, Central South University, Changsha, Hunan 410013, China

^9^National Clinical Research Center for Geriatric Disorders, Xiangya Hospital, Changsha, Hunan 410008, China

***Correspondence:** huixie@csu.edu.cn (Dr. Hui Xie); wangzx@csu.edu.cn (Dr. Zhen-Xing Wang); 600787@csu.edu.cn (Dr. Yong Zhou).

**Supplementary Materials**

The file includes:

Figs. S1 to S15

Tables 1 to 3


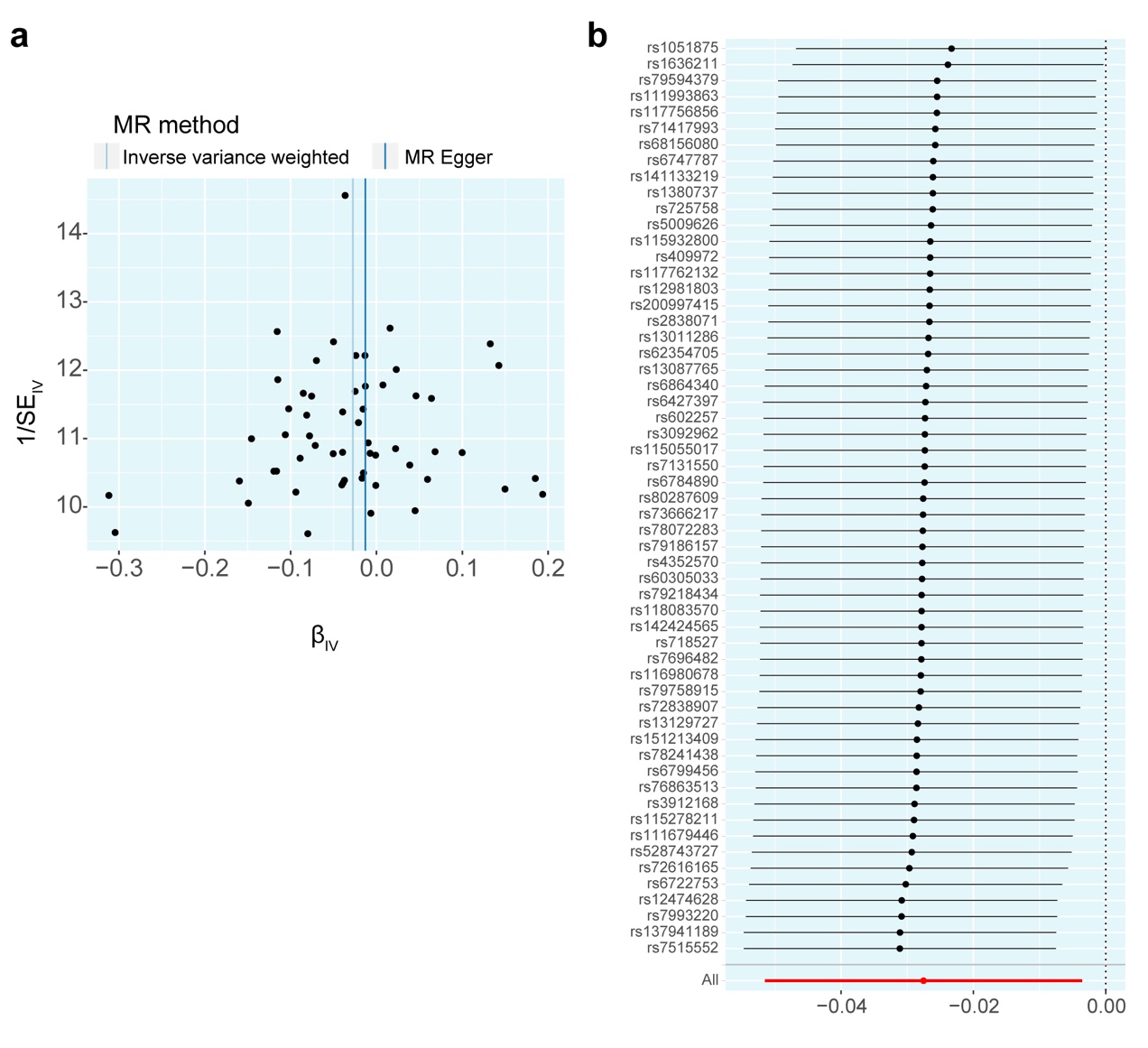


**Fig. S1. Sensitivity analyses for the association between intestinal *Akk* abundance and IVDD risk.**

**a** Funnel plot demonstrating the absence of heterogeneity among SNPs.

**b** Leave-one-out analysis showing that no single SNP unduly influenced the MR results.

**
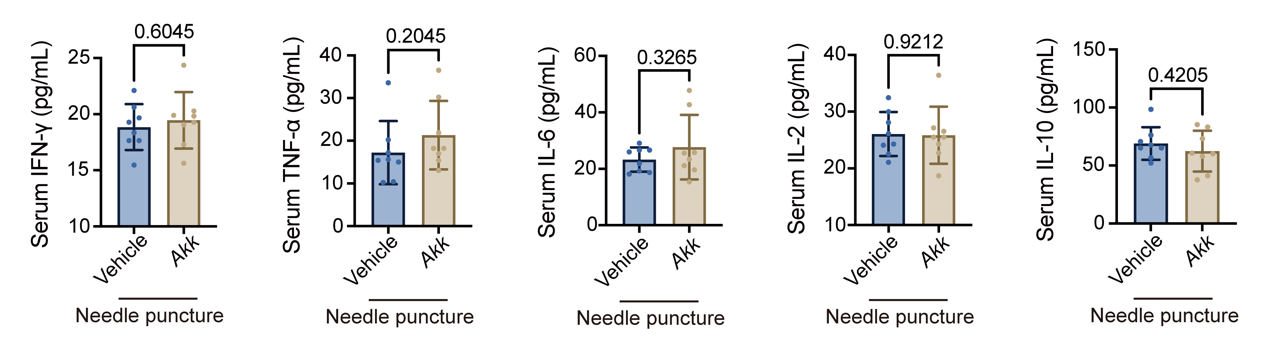
**

**Fig. S2. *Akk* supplementation does not induce systemic inflammation during the 8-week intervention in the needle puncture model.**

Quantitative analysis of serum inflammatory cytokines. n = 8 per group

Data are presented as mean ± SD. Statistical significance was determined by unpaired Student’s t-test.


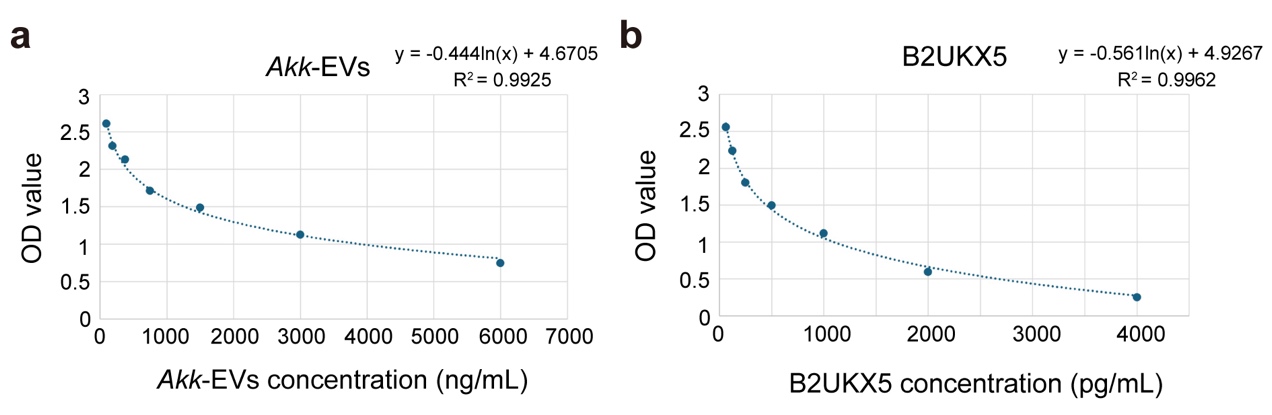


**Fig. S3.** **Validation of competitive ELISA for quantification of *Akk*-EVs and B2UKX5.**

**a-b** ELISA standard curves of *Akk*-EVs (a) and B2UKX5 (b) for spiked serum samples.


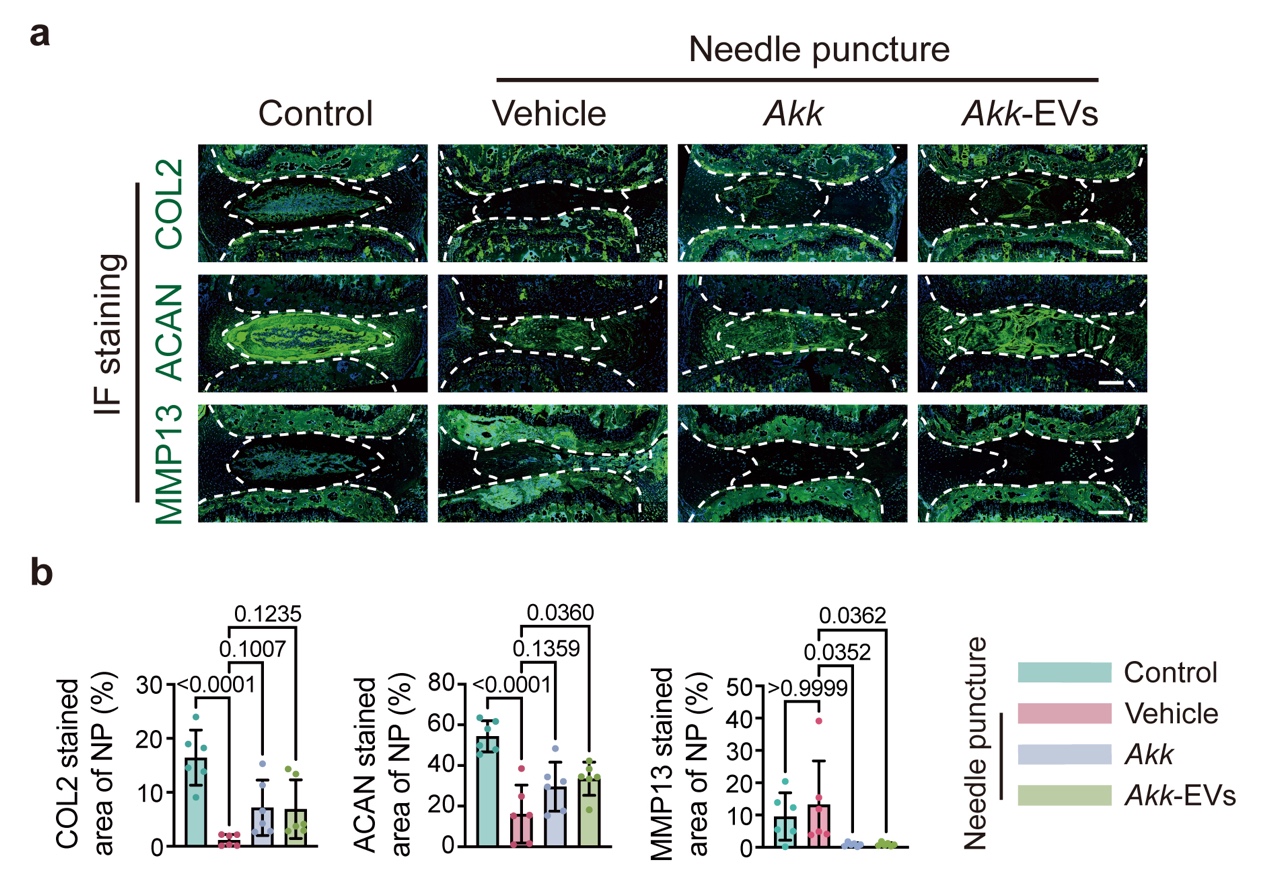


**Fig. S4. Orally administered *Akk* and *Akk*-EVs maintain ECM integrity in the tail needle puncture model.**

**a** Representative IF images of COL2, ACAN, and MMP13 expression in the C8-9 caudal segment. Scale bar: 200 μm.

**b** Quantitative analysis of IF staining for COL2, ACAN, and MMP13 in the C8-9 caudal segment. n = 6 per group.

Data are presented as mean ± SD. Statistical significance was assessed by one-way ANOVA followed by Bonferroni post hoc test.


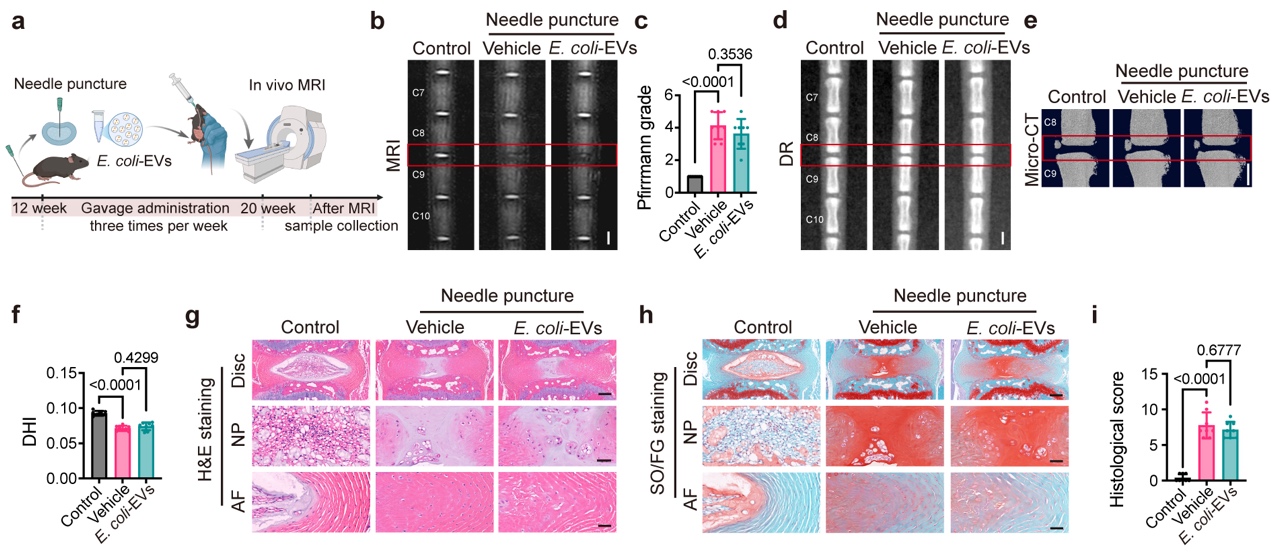


**Fig. S5.** **Orally administered *E. coli*-EVs do not alleviate IVDD in needle puncture model.**

**a** Schematic of the experimental protocol for *E. coli*-EVs treatment via oral gavage in tail needle puncture mice.

**b** Representative T2-weighted MRI images of the C8-9 caudal spine. Scale bar: 1 mm.

**c** Statistical analysis of Pfirrmann grading for the C8-9 caudal segment. n = 8 per group.

**d-e** Representative DR images (d) and micro-CT 3D reconstruction images (e) of the caudal spine. Scale bar: 1 mm.

**f** DHI percentage after 8 weeks of needle puncture at the C8-9 caudal segment. n = 8 per group.

**g-h** Representative H&E (g) and SO/FG (h) stained images showing the macroscopic morphology of the disc, NP, and AF in the C8-9 segment. Scale bars: 200 μm (Disc) or 50 μm (NP, AF).

**i** Histological scores for the C8-9 segment. n = 8 per group.

Data are presented as mean ± SD. Statistical significance was assessed by one-way ANOVA followed by Bonferroni post hoc test.


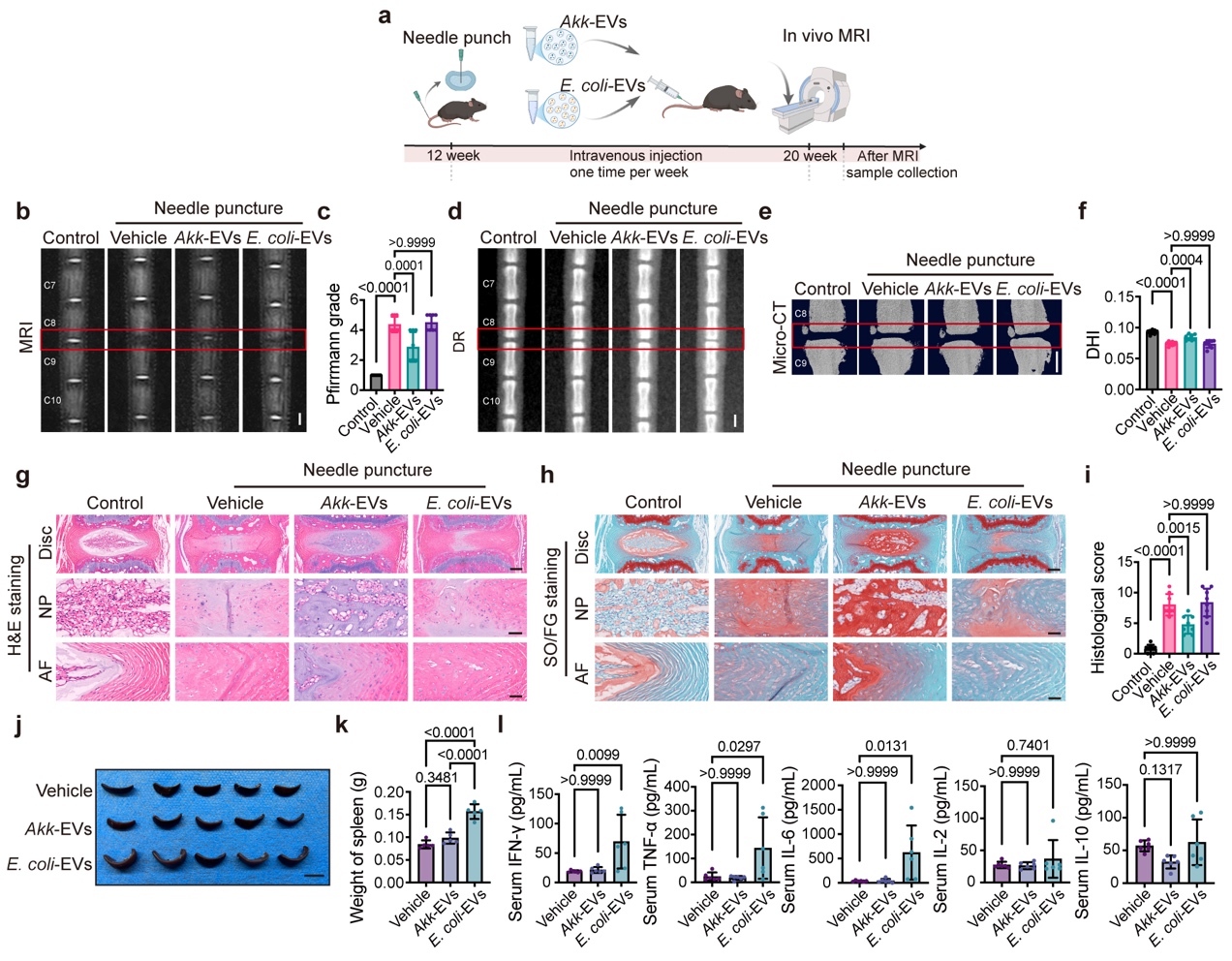


**Fig. S6. *Akk*-EVs, but not *E. coli*-EVs, protect against IVDD and do not induce systemic inflammation following tail vein administration.**

**a** Schematic of the experimental protocol for *Akk*-EVs and *E. coli*-EVs treatment via tail vein injection in tail needle puncture mice.

**b** Representative T2-weighted MRI images of the C8-9 caudal spine. Scale bar: 1 mm.

**c** Statistical analysis of Pfirrmann grading for the C8-9 caudal segment. n = 8 per group.

**d-e** Representative DR images (d) and micro-CT 3D reconstruction images (e) of the caudal spine. Scale bar: 1 mm.

**f** DHI percentage after 8 weeks of needle puncture at the C8-9 caudal segment. n = 8 per group.

**g-h** Representative H&E (g) and SO/FG (h) stained images showing the macroscopic morphology of the disc, NP, and AF in the C8-9 segment. Scale bars: 200 μm (Disc) or 50 μm (NP, AF).

**i** Histological scores for the C8-9 segment. n = 8 per group.

**j** Representative gross morphology of the spleen. Scale bars: 1 cm

**k** Quantification of spleen weight. n = 5 per group.

**l** Quantitative analysis of serum inflammatory cytokines. n = 6 per group

Data are presented as mean ± SD. Statistical significance was assessed by one-way ANOVA followed by Bonferroni post hoc test.


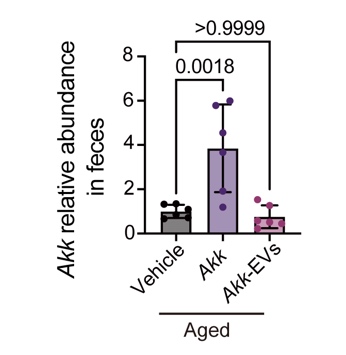


**Fig. S7.** **Successful colonization of *Akk* in the natural aging model following oral supplementation.**

qRT-PCR analysis of fecal *Akk* abundance in naturally aging mice treated with vehicle, *Akk*, or *Akk*-EVs. n = 6 per group.

Data are presented as mean ± SD. Statistical significance was determined by unpaired Student’s t-test.


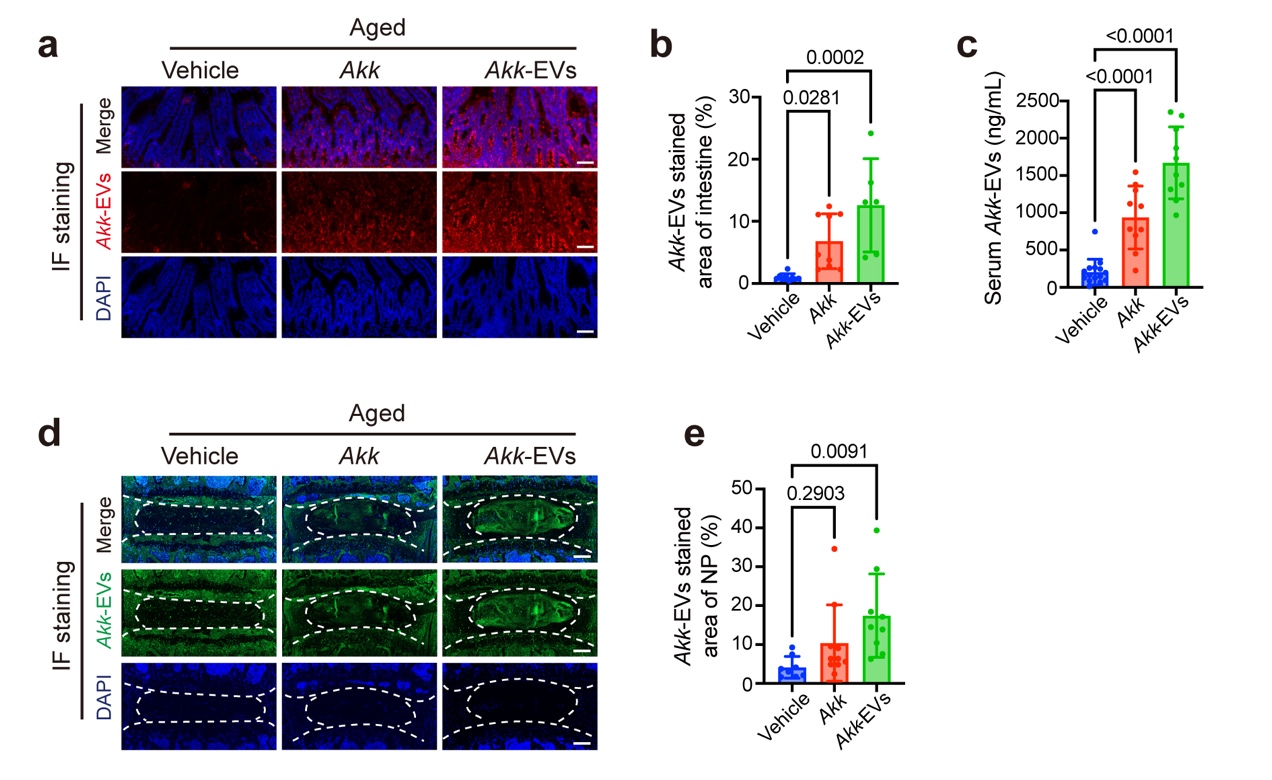


**Fig. S8.** **Elevated** ***Akk*-EVs abundance in intestine, circulation, and intervertebral discs following oral *Akk* and *Akk*-EVs supplementation.**

**a** Representative IF staining for *Akk*-EVs (red) in mouse gut tissue after treatment; Nuclei are counterstained with DAPI (blue). Scale bar: 50 μm.

**b** Quantification of *Akk*-EVs-positive area (%) in mouse gut tissue. n = 6-9 per group.

**c** Quantitative analysis of serum *Akk-*EVs levels in mice treated with Vehicle, *Akk*, or *Akk*-EVs. n = 10-15 per group.

**d** Representative IF staining for *Akk*-EVs (red) in mouse intervertebral disc tissue after treatment; Nuclei are counterstained with DAPI (blue). Scale bar: 200 μm.

**e** Quantification of *Akk*-EVs-positive area (%) in mouse intervertebral disc tissue. n = 8-10 per group.

Data are presented as mean ± SD. Statistical significance was assessed by one-way ANOVA followed by Bonferroni post hoc test.

**
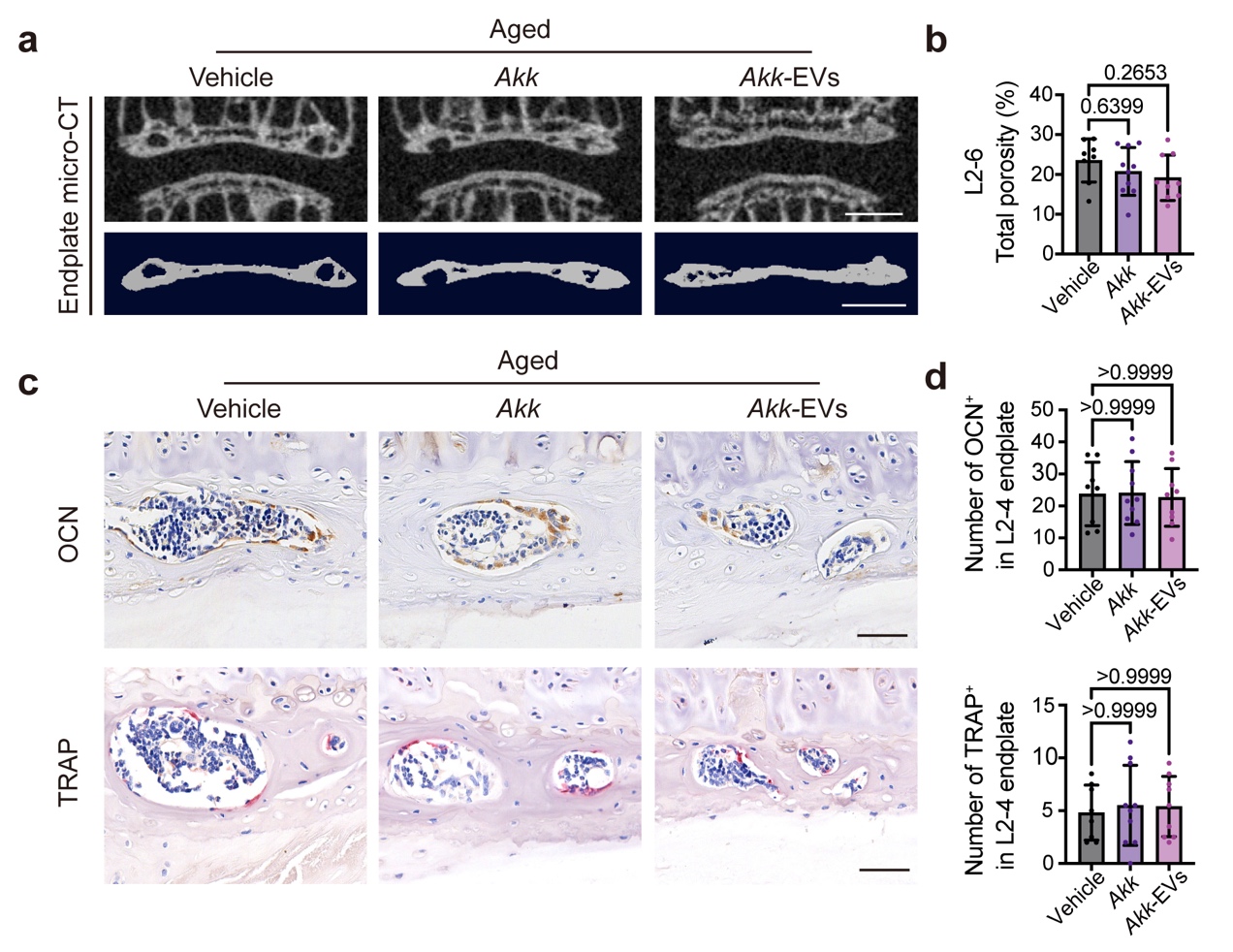
**

**Fig. S9. *Akk* and *Akk*-EVs do not significantly alter endplate porosity or bone remodeling activity in the natural aging mouse model.**

**a** Representative micro-CT 3D reconstruction images of the L3-4 endplate. Scale bar: 500 μm.

**b** Statistical analysis of the mean total porosity in the L2-6 lumbar disc segments. n = 8-10 per group.

**c** Representative immunohistochemical images of OCN and TRAP staining in the endplate. Scale bar: 50 μm.

**d** Quantitative analysis of OCN-positive and TRAP-positive cell numbers in the L2-4 lumbar disc segments. n = 8-10 per group.

Data are presented as mean ± SD. Statistical significance was assessed by one-way ANOVA followed by Bonferroni post hoc test.

**
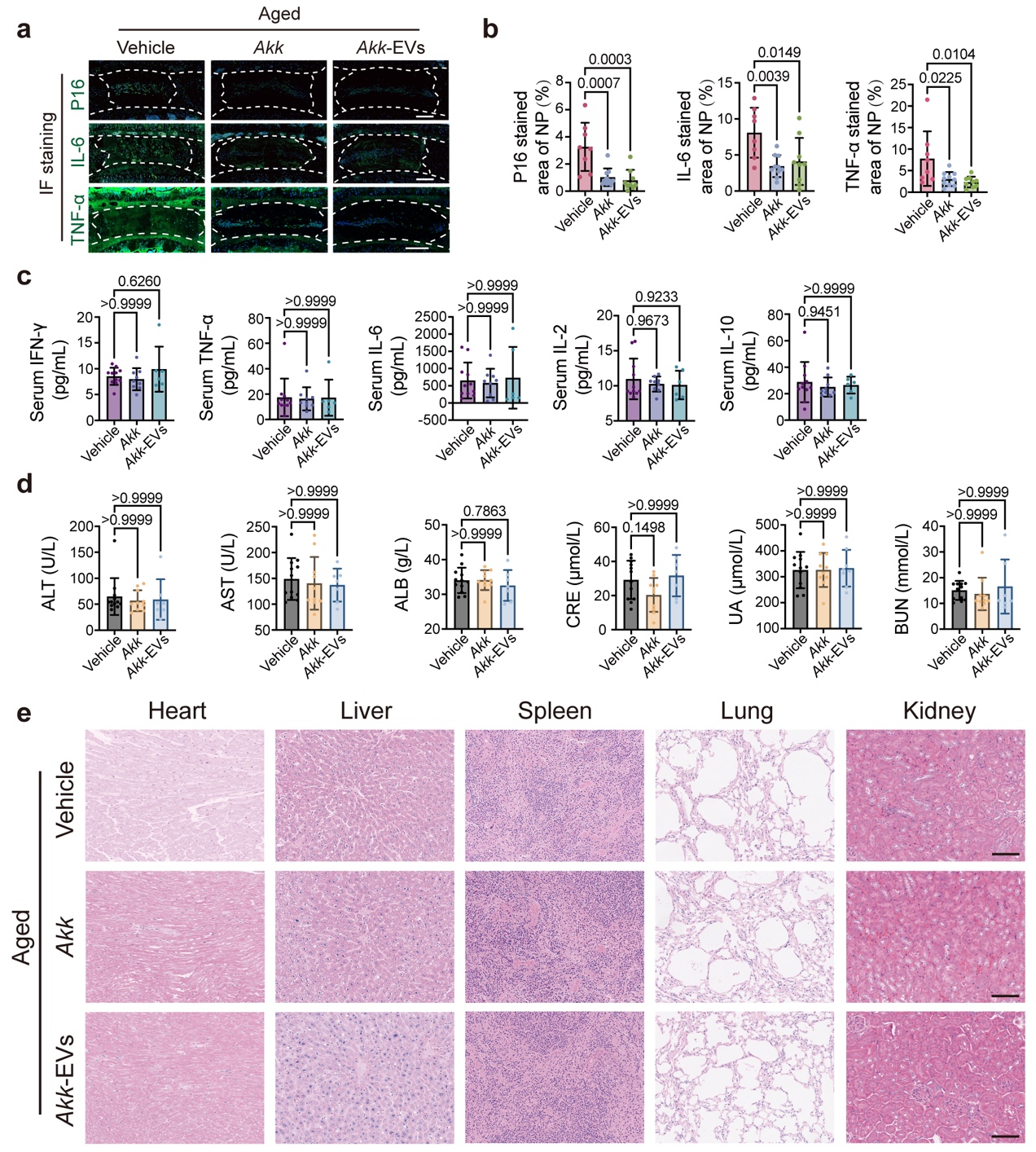
**

**Fig. S10.** ***Akk* and *Akk*-EVs alleviate disc inflammation and senescence without inducing systemic inflammation or organ toxicity.**

**a** Representative IF images showing the expression of p16^Ink4a^ (L5-6), IL-6 (L5-6) and TNF-α (L2-3) in the lumbar intervertebral disc. Scale bar: 200 μm.

**b** Quantitative analysis of p16^Ink4a^, IL-6 and TNF-α expression. n = 8-10 per group.

**c** Quantitative analysis of serum inflammatory cytokines. n = 6-11 per group.

**d** Serum biochemical analysis for liver and kidney function in treated mice. n = 7-12 per group.

**e** Representative H&E staining of major organs. Scale bar: 100 μm.

Data are presented as mean ± SD. Statistical significance was assessed by one-way ANOVA followed by Bonferroni post hoc test.

**
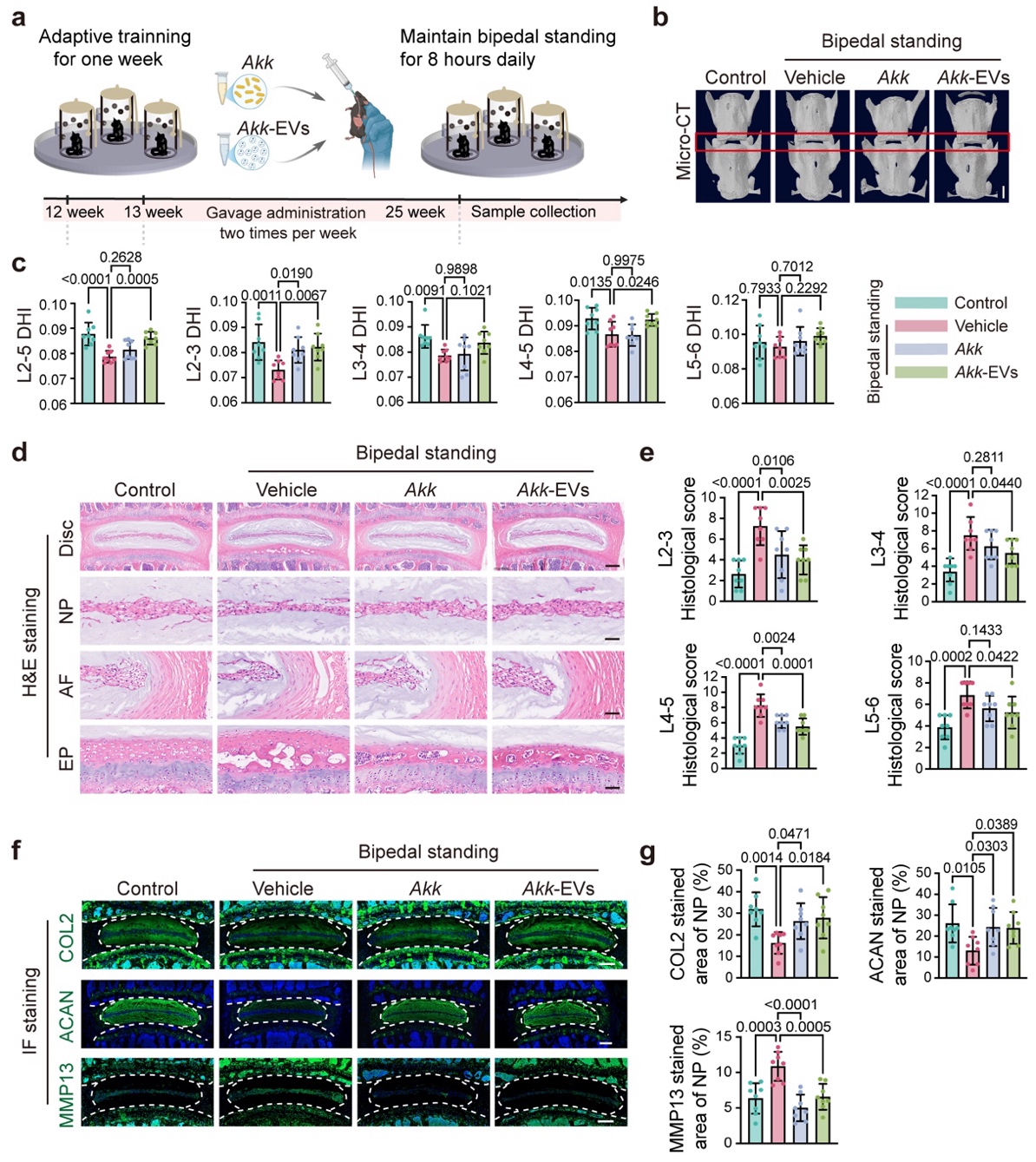
**

**Fig. S11. Supplementation of *Akk* and *Akk*-EVs attenuates mechanically induced IVDD in the bipedal standing model.**

**a** Schematic of the experimental protocol for the bipedal standing model.

**b** Representative micro-CT 3D reconstruction images of the L2-3 lumbar disc segments. Scale bar: 1 mm.

**c** DHI analysis of the average value in the L2-5 segments or each value for the individual segment in L2-6. n = 8 per group.

**d** Representative H&E-stained images showing the macroscopic morphology of the disc, NP, AF, and EP in the L4-5 segment. Scale bars: 200 μm (Disc) or 50 μm (NP, AF, EP).

**e** Histological scores for the L2-6 segments. n = 8 per group.

**f** Representative IF images of COL2, ACAN, and MMP13 expression in the L4-5 lumbar segment. Scale bar: 200 μm.

**g** Quantitative analysis of IF staining for COL2, ACAN, and MMP13. n = 8 per group.

Data are presented as mean ± SD. Statistical significance was assessed by one-way ANOVA followed by Bonferroni post hoc test.

**
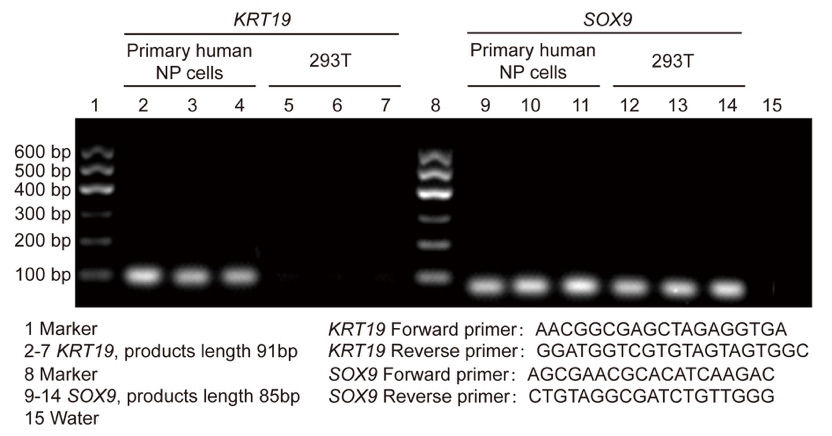
**

**Fig. S12. Cell identification.**

Identification of human primary NP cells by PCR analysis using markers *KRT19* and *SOX9*. 293T cells were used as control.

**
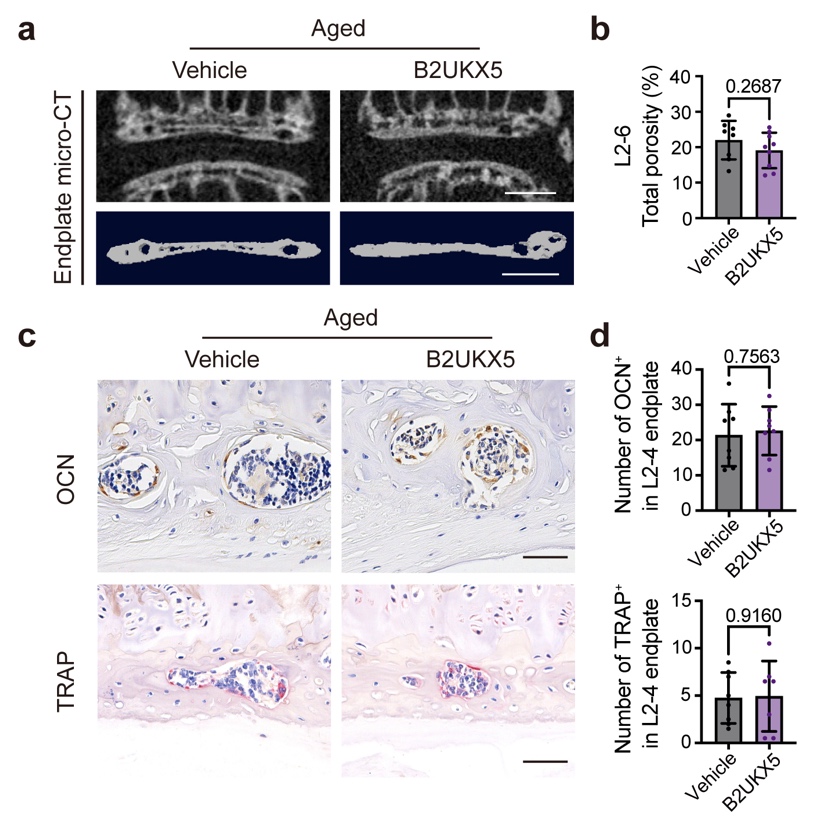
**

**Fig. S13.** **B2UKX5 does not significantly alter endplate porosity or bone remodeling activity in the natural aging mouse model.**

**a** Representative micro-CT 3D reconstruction images of the L3-4 endplate. Scale bar: 500 μm.

**b** Statistical analysis of the mean total porosity in the L2-6 lumbar disc segments. n = 8-9 per group.

**c** Representative immunohistochemical images of OCN and TRAP staining in the endplate. Scale bar: 50 μm.

**d** Quantitative analysis of OCN-positive and TRAP-positive cell numbers in the L2-4 lumbar disc segments. n = 7-8 per group.

Data are presented as mean ± SD. Statistical significance was determined by unpaired Student’s t-test.

**
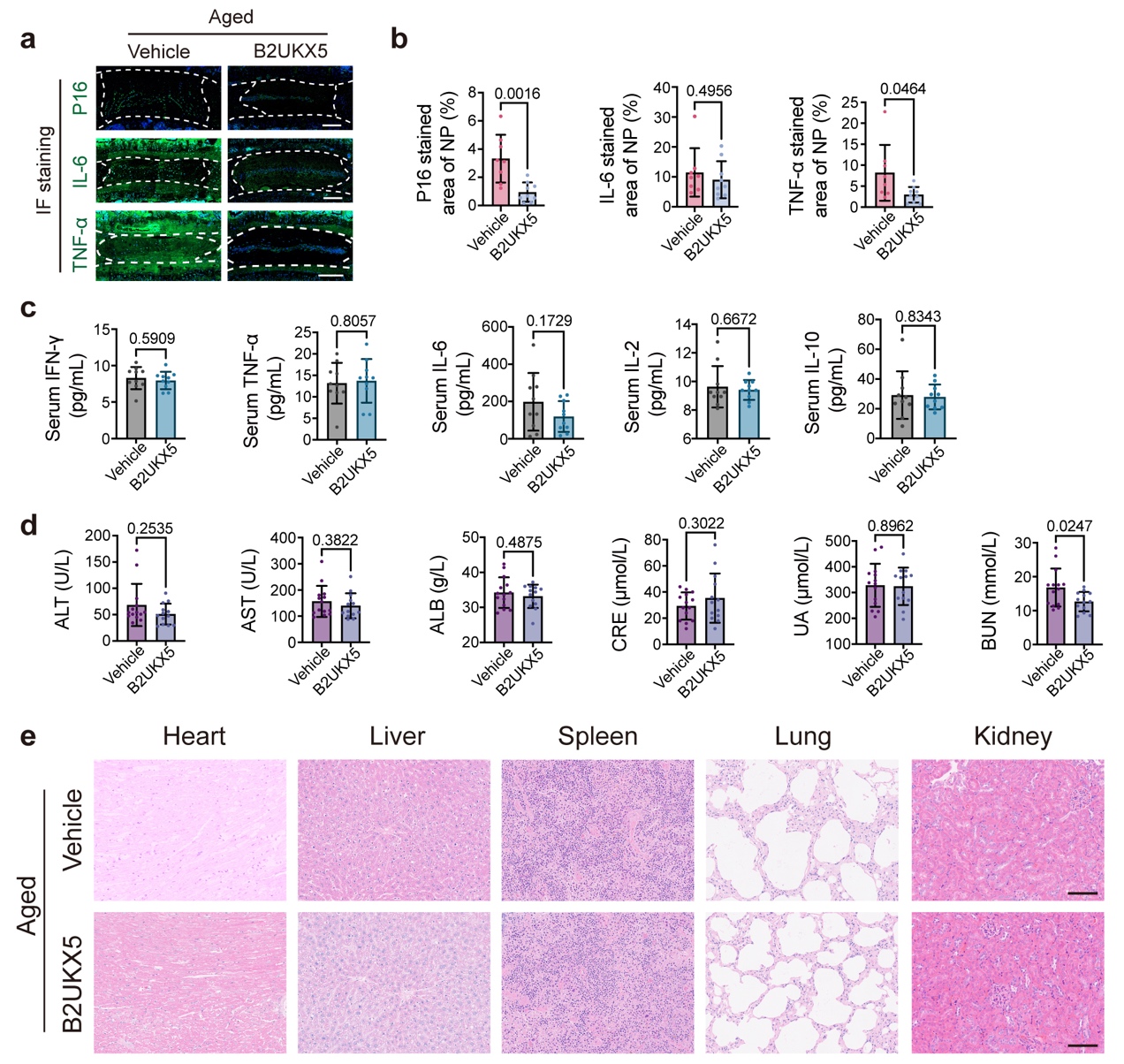
**

**Fig. S14. B2UKX5** **alleviates disc inflammation and senescence without inducing systemic inflammation or organ toxicity.**

**a** Representative IF images showing the expression of p16^Ink4a^ (L5-6), IL-6 (L5-6) and TNF-α (L2-3) in the lumbar intervertebral disc. Scale bar: 200 μm.

**b** Quantitative analysis of p16^Ink4a^, IL-6 and TNF-α expression. n = 8-9 per group.

**c** Quantitative analysis of serum inflammatory cytokines. n = 10 per group.

**d** Serum biochemical analysis for liver and kidney function in treated mice. n = 10-14 per group.

**e** Representative H&E staining of major organs. Scale bar: 100 μm.

Data are presented as mean ± SD. Statistical significance was assessed using Student’s t-test.

**
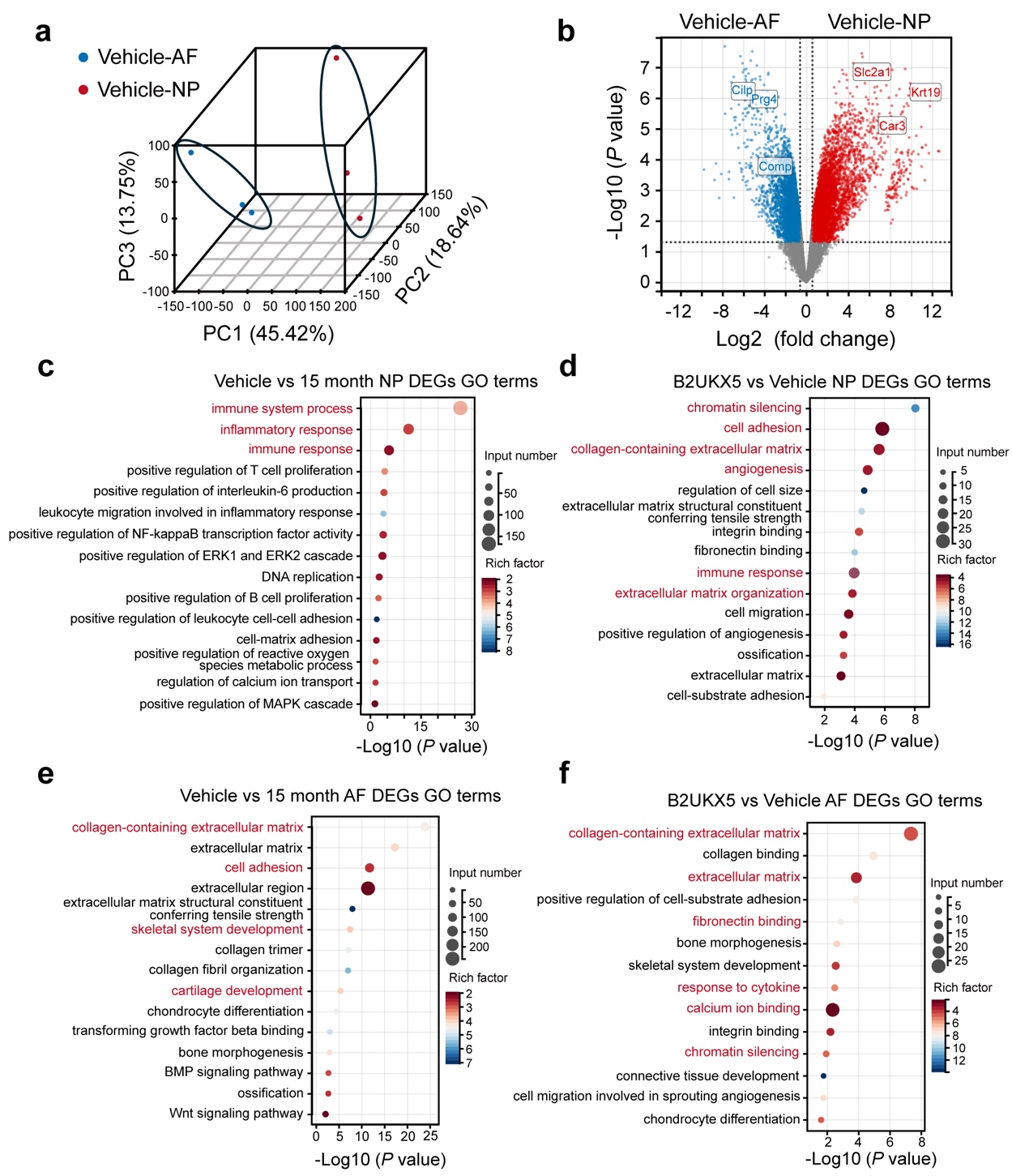
**

**Fig. S15. Transcriptomic quality control and GO enrichment analyses of NP and AF tissues.**

**a** Principal component clustering analysis of transcriptome sequencing data from NP and AF tissues in the vehicle-treated group.

**b** Volcano plot showing DEGs between NP and AF tissues.

**c** GO enrichment analysis of DEGs in NP tissue between the vehicle-treated and 15-month-old groups.

**d** GO enrichment analysis of DEGs in NP tissue between B2UKX5-treated and vehicle-treated groups.

**e** GO enrichment analysis of DEGs in AF tissue between the vehicle and 15-month-old groups.

**f** GO enrichment analysis of DEGs in AF tissue between B2UKX5-treated and vehicle-treated groups.

**Supplementary Table 1.** **Clinical sample information for cohort 1.**

| ID | Gender | Age | Average Pfirrmann grade (L1-S1) | *Akk* relative abundance  in feces |
| --- | --- | --- | --- | --- |
| 1 | Male | 62 | 3.2 | 0.78953 |
| 2 | Male | 54 | 2.8 | 7.70468 |
| 3 | Female | 76 | 4 | 0.35251 |
| 4 | Male | 50 | 3.2 | 2.82008 |
| 5 | Female | 62 | 3.8 | 0.00449 |
| 6 | Male | 55 | 3.2 | 0.00184 |
| 7 | Male | 61 | 2.4 | 0.00066 |
| 8 | Male | 60 | 3.4 | 1.38102 |
| 9 | Female | 67 | 3.8 | 1.66525 |
| 10 | Male | 55 | 3.2 | 0.00013 |
| 11 | Female | 76 | 4.6 | 0.67474 |
| 12 | Female | 60 | 3 | 6.91935E-06 |
| 13 | Female | 58 | 3.2 | 0.00037 |
| 14 | Female | 69 | 3.6 | 1.67702E-07 |
| 15 | Male | 75 | 3.8 | 0.00155 |
| 16 | Female | 12 | 1.2 | 5.78534 |
| 17 | Male | 35 | 2.4 | 5.92741 |
| 18 | Male | 45 | 2.8 | 0.10775 |
| 19 | Male | 74 | 4 | 0.02860 |
| 20 | Female | 71 | 4.4 | 0.00263 |
| 21 | Female | 76 | 3.8 | 5.64629E-06 |
| 22 | Male | 72 | 3.6 | 0.03908 |
| 23 | Female | 62 | 2.2 | 1.25299E-05 |
| 24 | Female | 78 | 4 | 0.00051 |
| 25 | Male | 57 | 2.6 | 0.00121 |
| 26 | Female | 58 | 3.4 | 0.00080 |
| 27 | Male | 55 | 3.4 | 2.12246 |
| 28 | Female | 70 | 4.2 | 0.00015 |
| 29 | Male | 73 | 4 | 0.00033 |
| 30 | Male | 71 | 3.8 | 0.00206 |
| 31 | Female | 72 | 3.8 | 0.06481 |
| 32 | Male | 59 | 3 | 2.74931 |
| 33 | Male | 66 | 3.6 | 0.01347 |
| 34 | Male | 52 | 3.2 | 0.02921 |
| 35 | Male | 52 | 2.6 | 4.48695 |
| 36 | Male | 57 | 3.8 | 0.13497 |
| 37 | Female | 58 | 3 | 1.22751 |
| 38 | Male | 50 | 3.6 | 0.02975 |
| 39 | Female | 54 | 2.4 | 0.04900 |
| 40 | Male | 70 | 3.2 | 0.11001 |
| 41 | Male | 57 | 3 | 7.73844E-05 |
| 42 | Female | 74 | 4 | 0.13893 |
| 43 | Male | 71 | 3.4 | 0.07531 |
| 44 | Male | 62 | 3 | 0.40774 |
| 45 | Female | 59 | 3.8 | 0.07906 |
| 46 | Female | 49 | 3.4 | 0.11522 |
| 47 | Male | 58 | 2.6 | 0.09666 |
| 48 | Female | 73 | 4.2 | 0.05168 |
| 49 | Female | 62 | 3.8 | 0.11682 |
| 50 | Female | 68 | 3.8 | 0.01197 |
| 51 | Female | 51 | 3.8 | 7.11388E-06 |
| 52 | Female | 57 | 3 | 0.56608 |
| 53 | Male | 57 | 3.2 | 0.00012 |
| 54 | Male | 66 | 4 | 0.12039 |
| 55 | Female | 91 | 3.8 | 5.67797E-05 |
| 56 | Female | 41 | 3 | 8.35362 |
| 57 | Female | 60 | 3.4 | 2.00551E-06 |
| 58 | Male | 25 | 2.2 | 6.24370 |
| 59 | Male | 55 | 3.8 | 0.02941 |
| 60 | Female | 68 | 3.2 | 2.59435E-05 |
| 61 | Male | 47 | 2.6 | 2.07638 |
| 62 | Female | 65 | 3.4 | 0.00032 |

**Supplementary Table 2. Clinical sample information for cohort 2.**

| ID | Gender | Age | Average Pfirrmann grade (L1-S1) | *Akk*-EVs in serum (ng/mL) | B2UKX5 in serum (pg/mL) | Operative segment Pfirrmann grade | *Akk*-EVs’ area in NP (%) | B2UKX5’s area in NP (%) |
| --- | --- | --- | --- | --- | --- | --- | --- | --- |
| 1 | Male | 60 | 2.4 | 592.297 | 557.736 | 3 | 4.987 | 3.290 |
| 2 | Female | 61 | 4.8 | 250.548 | 152.625 | 5 | 1.283 | 0.960 |
| 3 | Female | 70 | 2.6 | 308.928 | 141.616 | 3 | 2.817 | 2.368 |
| 4 | Male | 74 | 4.2 | 420.593 | 82.665 | 5 | 3.313 | 1.497 |
| 5 | Male | 69 | 3.6 | 564.936 | 79.770 | - | - | - |
| 6 | Male | 63 | 3.8 | 493.527 | 519.353 | - | - | - |
| 7 | Male | 61 | 3.6 | 243.318 | 241.313 | - | - | - |
| 8 | Male | 63 | 2.4 | 538.838 | 200.123 | 3 | 5.552 | 6.077 |
| 9 | Female | 58 | 3.6 | 225.381 | 211.870 | 3 | 4.525 | 1.492 |
| 10 | Male | 58 | 2.6 | 257.411 | 368.174 | 4 | 2.449 | 1.006 |
| 11 | Male | 72 | 4 | 218.878 | 316.975 | - | - | - |
| 12 | Female | 57 | 3.8 | 258.574 | 616.284 | 4 | 1.918 | 1.105 |
| 13 | Female | 74 | 2.6 | 189.070 | 609.728 | - | - | - |
| 14 | Female | 57 | 3 | 400.261 | 621.801 | - | - | - |
| 15 | Male | 63 | 3.2 | 232.601 | 95.676 | - | - | - |
| 16 | Female | 73 | 3.2 | 222.857 | 425.362 | - | - | - |
| 17 | Female | 65 | 3.8 | 381.771 | 371.470 | 3 | 1.611 | 2.735 |
| 18 | Female | 52 | 3.4 | 520.938 | 326.146 |  |  |  |
| 19 | Female | 50 | 3 | 231.035 | 234.111 | 3 | 4.163 | 3.291 |
| 20 | Male | 71 | 3.8 | 206.429 | 230.385 | 5 | 1.178 | 1.388 |
| 21 | Female | 60 | 4.2 | 551.111 | 229.156 | 4 | 1.762 | 3.091 |
| 22 | Male | 73 | 3.2 | 265.060 | 205.180 | - | - | - |
| 23 | Female | 55 | 3.2 | 318.107 | 191.742 | 4 | 3.528 | 1.057 |
| 24 | Female | 60 | 3.8 | 564.936 | 190.719 | 5 | 0.607 | 1.977 |
| 25 | Female | 57 | 3 | 548.634 | 328.479 | 5 | 4.051 | 1.692 |
| 26 | Male | 51 | 2.8 | 483.623 | 609.728 | - | - | - |
| 27 | Female | 69 | 4 | 281.678 | 407.549 | - | - | - |
| 28 | Male | 54 | 3 | 509.336 | 336.180 | 3 | 2.716 | 2.945 |
| 29 | Female | 73 | 3.8 | 611.271 | 322.676 | - | - | - |
| 30 | Female | 74 | 4 | 338.051 | 282.800 | - | - | - |
| 31 | Female | 64 | 3.4 | 297.323 | 306.420 | - | - | - |
| 32 | Male | 70 | 3.6 | 679.527 | 227.528 | - | - | - |
| 33 | Male | 79 | 3.6 | 437.995 | 515.664 | - | - | - |
| 34 | Male | 70 | 3 | 674.951 | 87.987 | - | - | - |
| 35 | Male | 50 | 2.6 | 497.993 | 417.847 | - | - | - |
| 36 | Female | 55 | 2.2 | 280.412 | 406.823 | - | - | - |
| 37 | Female | 62 | 3 | 580.412 | 350.874 | - | - | - |
| 38 | Female | 51 | 2.6 | 264.463 | 432.241 | - | - | - |
| 39 | Female | 70 | 3.4 | 714.045 | 586.280 | - | - | - |
| 40 | Male | 55 | 2.8 | 394.000 | 238.321 | - | - | - |
| 41 | Female | 77 | 4 | 281.045 | 225.509 | - | - | - |
| 42 | Male | 55 | 3 | 251.679 | 467.507 | - | - | - |
| 43 | Female | 57 | 3 | 718.886 | 187.016 | - | - | - |
| 44 | Male | 57 | 2.6 | 371.591 | 159.865 | - | - | - |
| 45 | Male | 56 | 2.4 | 340.343 | 142.884 | - | - | - |
| 46 | Male | 69 | 3.4 | 523.290 | 206.280 | - | - | - |
| 47 | Male | 67 | 3.4 | 398.462 | 129.772 | - | - | - |
| 48 | Male | 57 | 4.2 | 544.940 | 111.725 | - | - | - |
| 49 | Male | 58 | 2.4 | 295.987 | 449.529 | - | - | - |
| 50 | Female | 65 | 3 | 740.246 | 368.174 | - | - | - |
| 51 | Female | 61 | 3.4 | 717.269 | 341.008 | - | - | - |
| 52 | Female | 56 | 2.6 | 643.771 | 260.073 | - | - | - |
| 53 | Male | 61 | 3 | 188.220 | 219.168 | - | - | - |
| 54 | Male | 73 | 3.4 | 483.623 | 705.693 | - | - | - |
| 55 | Female | 56 | 2.8 | 572.622 | 191.400 | - | - | - |
| 56 | Male | 76 | 3.4 | 469.668 | 124.115 | - | - | - |
| 57 | Male | 72 | 3.6 | 575.207 | 417.103 | - | - | - |
| 58 | Male | 65 | 3.6 | 231.743 | 265.696 | - | - | - |
| 59 | Male | 25 | 2.2 | 1769.768 | 2615.840 | - | - | - |
| 60 | Male | 19 | 2 | 3072.987 | 2759.530 | - | - | - |
| 61 | Male | 36 | 2.8 | 2442.252 | 1429.470 | - | - | - |
| 62 | Male | 25 | 2 | 2481.061 | 1736.030 | - | - | - |
| 63 | Male | 32 | 2.6 | 1574.175 | 1137.850 | - | - | - |
| 64 | Male | 37 | 2.6 | 2509.158 | 2864.790 | - | - | - |
| 65 | Male | 35 | 3 | 1143.292 | 752.462 | - | - | - |
| 66 | Male | 41 | 3.2 | 681.059 | 770.103 | - | - | - |
| 67 | Female | 43 | 3 | 1425.656 | 847.915 | - | - | - |
| 68 | Male | 48 | 3.2 | 856.949 | 735.226 | - | - | - |
| 69 | Male | 18 | 2.6 | 2143.178 | 822.606 | - | - | - |
| 70 | Female | 45 | 3.2 | 1777.758 | 568.780 | - | - | - |
| 71 | Male | 26 | 2.2 | 1359.796 | 720.950 | - | - | - |
| 72 | Male | 27 | 2.6 | 2167.449 | 589.423 | - | - | - |
| 73 | Male | 32 | 2.8 | 1428.871 | 296.216 | - | - | - |
| 74 | Female | 45 | 2.8 | 1110.302 | 404.653 | - | - | - |
| 75 | Female | 47 | 2.4 | 1781.766 | 402.495 | - | - | - |
| 76 | Male | 46 | 2.8 | 1381.404 | 1040.830 | - | - | - |
| 77 | Male | 25 | 2.2 | 3755.046 | 1109.810 | - | - | - |
| 78 | Male | 31 | 2.4 | 996.530 | 1540.600 | - | - | - |
| 79 | Male | 49 | 2.6 | 1563.574 | 945.312 | - | - | - |
| 80 | Female | 58 | - | - | - | 5 | 1.028 | 1.077 |
| 81 | Female | 58 | - | - | - | 5 | 1.392 | 1.153 |
| 82 | Female | 59 | - | - | - | 5 | 1.697 | 1.158 |
| 83 | Female | 65 | - | - | - | 5 | 1.098 | 1.256 |
| 84 | Female | 51 | - | - | - | 4 | 1.592 | 1.564 |
| 85 | Male | 57 | - | - | - | 4 | 2.732 | 1.038 |
| 86 | Female | 58 | - | - | - | 4 | 2.522 | 3.794 |
| 87 | Female | 60 | - | - | - | 4 | 2.092 | 1.570 |
| 88 | Female | 67 | - | - | - | 4 | 3.602 | 1.528 |
| 89 | Male | 53 | - | - | - | 3 | 6.312 | 3.387 |
| 90 | Male | 56 | - | - | - | 3 | 4.477 | 1.806 |
| 91 | Male | 61 | - | - | - | 3 | 4.469 | 2.294 |
| 92 | Male | 65 | - | - | - | 3 | 2.426 | 2.187 |

**Supplementary** **Table 3. Primer sequences.**

| **Gene** | **Forward (5’-3’)** | **Reverse (5’-3’)** |
| --- | --- | --- |
| *Universal* | ACTCCTACGGGAGGCAGCAGT | ATTACCGCGGCTGCTGGC |
| *Akk* | CCTTGCGGTTGGCTTCAGAT | CAGCACGTGAAGGTGGGGAC |
| *h-GAPDH* | AGAAAAACCTGCCAAATATGATGAC | TGGGTGTCGCTGTTGAAGTC |
| *h-MMP13* | ACTGAGAGGCTCCGAGAAATG | GAACCCCGCATCTTGGCTT |
| *h-ADAMTS5* | GAACATCGACCAACTCTACTCCG | CAATGCCCACCGAACCATCT |
| *h-TNF* | CCTCTCTCTAATCAGCCCTCTG | GAGGACCTGGGAGTAGATGAG |
| *h-CDKN1A* | AGGTGGACCTGGAGACTCTCAG | TCCTCTTGGAGAAGATCAGCCG |
